# Haplotyping SNPs for allele-specific gene editing of the expanded huntingtin allele using long-read sequencing

**DOI:** 10.1016/j.xhgg.2022.100146

**Published:** 2022-09-26

**Authors:** Li Fang, Alex Mas Monteys, Alexandra Dürr, Megan Keiser, Congsheng Cheng, Akhil Harapanahalli, Pedro Gonzalez-Alegre, Beverly L. Davidson, Kai Wang

**Affiliations:** 1 Raymond G. Perelman Center for Cellular and Molecular Therapeutics, Children’s Hospital of Philadelphia, Philadelphia, PA 19104, USA; 2 Sorbonne Université, Paris Brain Institute, AP-HP, INSERM, CNRS, University Hospital Pitié-Salpêtrière, Paris, France; 3 Huntington’s Disease Center and Division of Movement Disorders, Department of Neurology, The University of Pennsylvania, Philadelphia, PA 19104, USA; 4 Spark Therapeutics, Philadelphia, PA 19104, USA; 5 Department of Pathology and Laboratory Medicine, University of Pennsylvania Perelman School of Medicine, Philadelphia, PA 19104, USA

**Keywords:** Huntington’s disease, long-read sequencing, CRISPR, SNP, repeat detection

## Abstract

Huntington’s disease (HD) is an autosomal dominant neurodegenerative disease caused by CAG trinucleotide repeat expansions in exon-1 of *huntingtin* (*HTT*). Currently, there is no cure for HD, and the clinical care of individuals with HD is focused on symptom management. Previously, we showed allele-specific deletion of the expanded *HTT* allele (*mHTT)* using CRISPR-Cas9 by targeting nearby (<10 kb) SNPs that created or eliminated a protospacer adjacent motif (PAM) near exon-1. Here, we comprehensively analyzed all potential PAM sites within a 10.4-kb genomic region flanking exon-1 of *HTT* in 983 individuals with HD using a multiplex targeted long-read sequencing approach on the Oxford Nanopore platform. We developed computational tools (NanoBinner and NanoRepeat) to de-multiplex the data, detect repeats, and phase the reads on the expanded or the wild-type *HTT* allele. One SNP common to 30% of individuals with HD of European ancestry emerged through this analysis, which was confirmed as a strong candidate for allele-specific deletion of the *mHTT* in human HD cell lines. In addition, up to 57% HD individuals may be candidates for allele-specific editing through combinatorial SNP targeting. Cumulatively, we provide a haplotype map of the region surrounding exon-1 of *HTT* in individuals affected with HD. Our workflow can be applied to other repeat expansion diseases to facilitate the design of guide RNAs for allele-specific gene editing.

## Teaser

We sequenced 1,056 individuals with Huntington’s disease (HD), a neurodegenerative disease caused by repeat expansions in exon-1 of the gene, to identify candidate SNPs for allele-specific CRISPR-Cas9-mediated targeting. The resultant high-resolution haplotype map allowed for gRNA-targeting of the disease allele in patient cells.

## Introduction

Huntington’s disease (HD) (MIM: 143100) is an autosomal dominant neurodegenerative disease that affects 10.6–13.7 individuals per 100,000 in populations of European ancestry.^1^, ^2^, ^3^, ^4^ Individuals with HD suffer from progressive motor, cognitive, and psychiatric disturbances over the course of 10–20 years.^5^^,^^6^ At the molecular level, HD is caused by a CAG trinucleotide repeat expansion in exon-1 of the huntingtin (*HTT*, MIM: 613004) gene located at chromosome 4p16.3.^1^ In the normal population, the CAG repeat is in the range of 6–35.^2^ When expanded to ≥35 repeats, HD is likely to develop. In individuals with 36–39 repeats, there is partial penetrance^2^ with full penetrance when there are ≥40 repeats. The predicted rate by which an individual with CAG expansion will develop HD is determined by the longest expanded allele in a completely dominant manner.^7^

Currently, there is only symptomatic treatment for individuals with HD.^2^ Earlier studies using mouse models showed that HD-like phenotypes can be resolved if the expression of the expanded *HTT* (*mHTT*) allele is reduced, even at later disease stages,^8^, ^9^, ^10^ suggesting that reducing HTT expression, and in particular expanded HTT expression, may be clinically relevant.

Gene-silencing strategies using RNA interference (RNAi) or antisense oligonucleotides (ASOs) have been efficacious in mouse models.^9^, ^10^, ^11^ One trial done in collaboration between Ionis Pharmaceuticals and Roche approached non-allele selective silencing for HD individuals^12^ (ClinicalTrials.gov identifiers: NCT02519036 and NCT03342053). Phase III studies were ended early because participants receiving active drug progressed more rapidly than placebo-treated participants^13^ (ClinicalTrials.gov identifier: NCT03761849). In addition, two allele-selective ASOs were tested in individuals with HD (PRECISION-HD1 and PRECISION-HD2; ClinicalTrials.gov identifiers: NCT03225833 and NCT03225846) with results released recently. Unfortunately, neither ASO showed target engagement in cerebrospinal fluid. The PRECISION-HD2 core trial participants who received WVE-120102 (targeting rs362331) had a median reduction of 9.9% in *mHTT* in cerebrospinal fluid (p = 0.74) compared with the placebo group, who had a median decrease in *mHTT* of 0.8%. Results of the PRECISION-HD1 core trial were similar.^14^ Thus, it is important to develop and test additional gene-silencing strategies.

The recently discovered CRISPR-Cas9 system^15^, ^16^, ^17^ is a promising genome editing technology for genetic disorders such as HD. In this system, the Cas9 protein is co-expressed with single guide RNAs (sgRNAs) that together form a ribonucleoprotein complex (sgRNA-Cas9 complex) that binds specific genomic DNA sequences and mediates a double-strand DNA (dsDNA) break. Targeted gene deletions can be made by non-homologous end-joining when a pair of sgRNA-Cas9 complexes bind either end of the DNA target and produce dsDNA breaks. Targeting specificity of the sgRNA-Cas9 complex is regulated by two factors: (1) the binding affinity of the 20-nt sgRNA with the complementary genomic DNA sequence; and (2) the recognition of a protospacer adjacent motif (PAM) immediately following the genomic DNA/sgRNA complementary region.^15^^,^^16^ While mismatches on the sgRNA/DNA complementary sequence are tolerable, the presence of an intact PAM motif is critical, and mutations on the PAM sequence cause ablation of cleavage.^18^^,^^19^ Therefore, allele-specific gene editing could be achieved by taking advantage of SNPs that either eliminate or create PAM sequences.

We^20^^,^^21^ and other groups^22^ previously reported allele-specific editing of the *mHTT* allele *in vitro* and *in vivo*, taking advantage of SNPs identified for use with the CRISPR/SpCas9 system. However, these studies mainly screened common SNPs in the normal population and a detailed haplotype map surrounding exon-1 of the HD population is unknown. To more broadly adapt this approach to the HD population, we developed a robust, high-throughput pipeline to detect and phase all highly prevalent SNPs in the HD population that, when present, create a PAM *cis* to the expanded HTT allele that can be used together with the CRISPR systems. Notably, haplotype analysis of HD cohorts has been reported previously,^23^^,^^24^ with a focus on allele specific ASO or miRNA-based silencing strategies targeting the spliced and unspliced transcript. Given that the *HTT* gene is 170 kb in length, most SNPs analyzed were distant from exon-1, yet, for deletion of the CAG-repeat, a haplotype map surrounding exon-1 is required. Because we found earlier that editing efficacy reduces with increasing distance from exon-1,^20^ we focused our analysis within 10 kb of exon-1.

To address this problem, we used Oxford Nanopore long-read sequencing paired with two novel computational tools (NanoBinner and NanoRepeat) to de-multiplex data, detect repeat size, and haplotype the allele. This was applied to 319 samples from the French HD consortium and validated on 664 samples from the CHDI Foundation, which consists of DNA samples from multiple continents and ethnic groups. We identified all common SNPs in the French and CHDI HD cohorts, and analyzed all SNPs that provide PAMs to mediate CRISPR editing of the expanded *HTT* allele.

## Material and methods

### HD subjects

HD subjects from the French cohort were recruited at the Pitié Salpetrière Hospital in Paris, France. All subjects gave written informed consent, and blood samples were collected in accordance with local French regulations (Paris Necker ethics committee approval [RBM 03-48] to A.D.). Genomic DNA samples from the CHDI cohort were generously provided by the participants in the Enroll-HD study and made available by the CHDI Foundation. Detailed information of the HD subjects (including race, sex, region, and CAG repeat sizes) is shown in Tables S11 and S12. Enroll-HD is a global clinical research platform intended to accelerate progress toward therapeutics for HD; certain samples and core datasets are collected annually on all research participants as part of this multi-center longitudinal observational study. Enroll-HD is sponsored by the CHDI Foundation, a nonprofit biomedical research organization exclusively dedicated to developing therapeutics for HD.

### Barcode design for multiplexed long-read sequencing

The barcode design workflow used in this study is shown in Figure S1A. We designed our in-house barcode sequences, which met the following criteria: (1) there was no homopolymer that was longer than 2 bp (e.g, “TT” was allowed but “TTT” was not allowed); (2) there was no tandem repeat; (3) GC content is within 40%–60%; (4) the barcode sequence cannot be mapped to human reference genome GRCh38 so that its binding with human genomic DNA during the PCR process is minimized; (5) pairwise sequence similarity between every two barcodes was minimized. To meet this goal, we first generated an excessive amount (20,000) of barcode sequences that meet criteria 1–3. Next, we aligned the barcode sequences to the GRCh38 reference genome using the blastn algorithm,^25^^,^^26^ and removed any barcodes aligned to GRCh38. Then we used a graph-based algorithm to find a set of barcodes where there was no pairwise alignment (blastn algorithm, word size = 6) between any two barcodes (Figure S1B). Each barcode was a node in the graph. Initially, all nodes are connected in the undirected graph. We performed an all-vs-all alignment of the barcode sequences using the blastn algorithm (word size = 6). We removed the edge between two barcodes (nodes) if the two barcodes were aligned. The remaining edges only connect barcode pairs that have no alignment. Therefore, a complete subgraph (clique) is a set of barcodes in which any two barcodes have no alignment. We used the networkx python package to find the cliques with enough nodes (number of barcodes). We first designed a set of 16-bp barcodes for amplicon-1 (Table S1; Figure S1). After analysis of the sequencing data of amplicon-1, we found that the first a few bases were trimmed in some of the reads and thus longer barcode sequences might be better for the de-multiplexing process. We designed 32-bp barcodes for amplicon-2 (Table S2) and amplicon-3 (Table S3).

### Barcoded long-range PCR to amplify the region flanking exon-1 of the *HTT* gene

**Figure 1:**
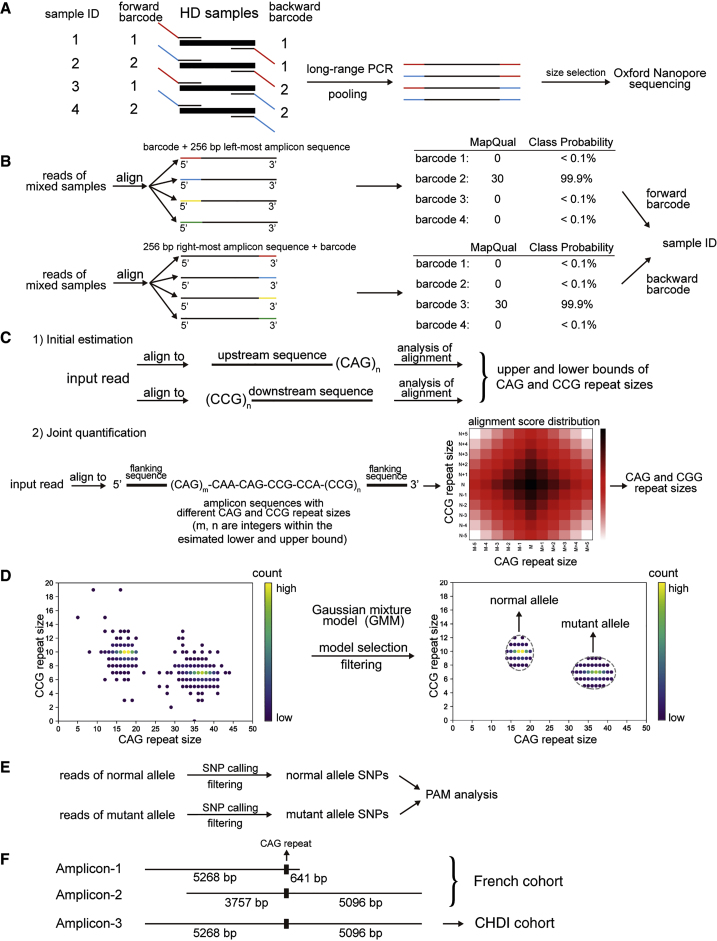
Sequencing and data analysis workflow (A) Barcodes were added to both forward and backward PCR primers and the pooled PCR products of multiple samples were sequenced with Oxford nanopore sequencing. (B) De-multiplexing strategy of NanoBinner. NanoBinner aligns a read to barcode sequences with the 256-bp amplicon sequence immediately next to it. The 256-bp amplicon sequence acts as an anchor so that the matching of barcodes is at the correct position. NanoBinner assigns a read to a barcode if the Phred scale mapping quality score is ≥30. (C) Joint quantification of CAG and CCG repeat sizes using NanoRepeat. NanoRepeat first estimates the upper and lower bounds of the CAG and CCG repeat sizes separately, and then performs a joint quantification to refine the repeat sizes. In the joint quantification step, NanoRepeat generates a series of template sequences with *m* CAG repeats and *n* CCG repeats, where *m* and *n* are all integers within the upper and lower bounds determined in the first step. A read was aligned to this series of template sequences. The CAG and CCG repeat sizes that maximize the alignment score was the final estimates of the repeat sizes of the read. (D) NanoRepeat separates reads using GMM. CAG and CCG repeat sizes are used as input features. The scatterplot shows the CAG and CCG repeat sizes of a typical example. The color of the points indicates the number of reads. Model selection is performed to select the best number of Gaussian models. After filtering outliers, the two alleles are well separated (right). The dashed gray circles are equi-probability surfaces of the fitted Gaussian models where the probability outside the surface is less than 5%. (E) SNPs detection was performed using longshot. Low quality SNP calls were removed. The effects of on PAMs were examined. (F) Locations of the PCR amplicons of each cohort. The lengths are the distance to the first nucleotide of the CAG repeat (based on GRCh38).

For the French HD cohort, the target region was covered by two overlapping amplicons (amplicon-1 and amplicon-2; Figure 1F). Amplicon-1 mainly covers the upstream region of the CAG repeat region (GRCh38, chr4:3069608-3075517). The amplicon length (without barcode) is 5,910 bp. Amplicon-2 mainly covers the downstream region of the CAG repeat region (GRCh38, chr4:3071119-3079972). The amplicon length (without barcode) is 8,854 bp. For each amplicon, ten different barcodes were added to the 5′ side of both forward and backward primers. The complete primer sequences are listed in Tables S1 and S2. The combination of the both forward and backward primers can be used to multiplex 100 samples. The French HD cohort contains 396 HD genomic DNA samples which were stored in five 96-well plates where plates 1–4 each contain 95 samples and plate 5 contains 16 samples. For each plate of plates 1–4, we performed barcoded PCR with 95 barcode combinations, and the pooled samples were then sequenced in one Nanopore flow cell (described below). Samples in plate 5 were sequenced together with the QC-failed samples of plates 1–4. The long-range PCR was performed using the PrimeSTAR GXL DNA Polymerase (Takara Bio USA). The PCR conditions are: (1) 95°C for 3 min; (2) 98°C for 10 s; (3) 68°C for 10 min. Steps 2 to 3 were repeated for 30 cycles.

For the CHDI HD cohort, the target region was covered by one single amplicon (amplicon-3; Figure 1F). The forward primer is the forward primer of amplicon-1 and the backward primer is the backward primer of amplicon-2. The amplicon length (without barcode) is 10,365 bp. Twenty-four different barcodes were added to the 5′ side of both forward and backward primers. The combination of both forward and backward primers can be used to multiplex 576 samples. The CHDI HD cohort contains 960 samples, which were stored in ten 96-well plates. We multiplex 480 samples (five plates) at a time. The PCR condition and sequencing process are the same as the French HD cohort.

### Long-read sequencing of the barcoded PCR products

The barcoded PCR products were pooled and purified with Agencourt AMPure XP beads (Beckman Coulter, A63881). The sequencing library was prepared using a ligation sequencing kit (Oxford Nanopore Technology [ONT], SQK-LSK109), according to manufacturer’s instructions.

(1) DNA repair, end repair, and dA-tailing: 1 μg of the pooled and purified PCR products was used as input DNA, and the volume was adjusted to 47 μL with nuclease-free water. One microliter of DNA CS (ONT, SQK-LSK109), 47 μL input DNA, 3.5 μL NEBNext FFPE DNA Repair Buffer (NEB, M6630), 2 μL NEBNext FFPE DNA Repair Mix (NEB, M6630), 3.5 μL Ultra II End-prep reaction buffer (NEB, E7546), and 3 μL Ultra II End-prep enzyme mix (NEB, E7546) were mixed in a PCR tube. The mixture was incubated at 20°C for 5 min and 65°C for 5 min. A 1× volume (60 μL) AMPure XP clean-up was performed, and the DNA was eluted in 61 μL nuclease-free water. A 1-μL aliquot was quantified by fluorometry (Qubit) to ensure ≥700 ng DNA was retained.
(2) Adapter ligation: 60 μL DNA sample from the previous step, 25 μL Ligation Buffer (ONT, SQK-LSK109), 10 μL NEBNext Quick T4 DNA Ligase (NEB, E6056), and 5 μL Adapter Mix (ONT, SQK-LSK109) were mixed in order. The mixture was incubated for 10 min at room temperature. The adaptor-ligated DNA was cleaned up by adding a 0.4× volume (40 μL) of AMPure XP beads, incubating for 5 min at room temperature, and resuspending the pellet twice in 250 μL Long Fragment Buffer (ONT, SQK-LSK109). The purified-ligated DNA was resuspended in 15 μL elution buffer (ONT, SQK-LSK109). A 1-μL aliquot was quantified by fluorometry (Qubit) to ensure ≥300 ng DNA was retained. A total of 50 fmol (278 ng) of this final prepared library was loaded onto the GridION sequencer with an FLO-MIN106D (R9.4.1) flow cell. The sequencing was run for 48 h. Basecalling was performed using the super-accuracy model of Guppy (v.5.0.14).

### Demultiplexing of the sequencing data with NanoBinner

We developed NanoBinner, a tool for de-multiplexing of barcoded amplicons from long-read sequencing data. Given the moderate error rate in long reads, there might be random matches of barcodes inside the amplicon sequence. To avoid this potential issue, NanoBinner aligns the barcode sequence as well as the 256-bp amplicon sequence next to it. The 256-bp amplicon sequence acts as an anchor so that the matching of barcodes is at the correct position. The alignment is performed using minimap2^27^ with the parameter for nanopore reads (-x map-ont). This parameter can be changed if the input is PacBio reads. Minimap2 calculates a mapping quality score for each barcode, which is the Phred scale of the probability that a read is misplaced. NanoBinner assigns a read to a barcode if the Phred scale mapping quality score is ≥30. In our case, the combination of the two barcodes on both sides determines the sample. A read was assigned to a specific sample if the barcodes on both sides were confidently determined. One FASTQ file was generated for each sample.

### Repeat detection and read phasing with NanoRepeat

NanoRepeat can quantify a single tandem repeat or jointly quantify two adjacent repeats. In this study, we jointly quantify the CAG and CCG repeats in the *HTT* gene. The joint quantification process has two steps: fast estimation and refining. In the fast estimation step, NanoRepeat performs a quick analysis of each repeat and estimates the lower and upper bound of the repeat size independently. This analysis is done by aligning the reads to a decoy reference sequence with 1,000 repeat units (CAG or CCG) using minimap2.^27^ Given the sequencing error rate, the alignment has some tolerance of mismatches and, thus, the nearby non-repeat region in the reads might be forced to be aligned with the decoy reference sequence. Therefore, the upper bound of the repeat size is the number of aligned repeat units in the decoy reference sequence. We assign the lower bound of the repeat size as the number of repeat units in the read that exactly matched the reference sequence with no error. Let *L*_*1*_, *L*_*2*_ denote the lower bounds of the CAG and CCG repeats, and *U*_*1*_, *U*_*2*_ denote the upper bounds of the two repeats, respectively. In the refining step, NanoRepeat generates a batch of amplicon sequences with *m* (*L*_*1*_ ≤ *m* ≤ *U*_*1*_) CAG repeat units and *n* (*L*_*2*_ ≤ *n* ≤ *U*_*2*_) CCG repeat units (Figure 1C). Each read is aligned to this batch of amplicon sequences with minimap2.^27^ The *m* and *n* that maximize the alignment score were the estimated CAG and CCG repeat sizes of the read.

After the repeat number of each read is determined, NanoRepeat classifies the reads to alleles. First, we remove outlier reads with repeat sizes that are outside three standard deviations from the mean. Next, we assume that the CAG and CCG repeat sizes (*m*, *n*) are distributed according to a mixture of *N* Gaussian models, where *N* is 1 or 2 as human is a diploid genome. Akaike information criterion or Bayesian information criterion are commonly used criteria to select the best value of *N* and prevent overfitting. As Gaussian distribution is a probability distribution of real-valued random variables, we found that these two methods are not able to prevent overfitting of the Gaussian mixture model (GMM) if the input random variables are rounded to integers. Therefore, we added a uniform distributed random noise (between −0.5 and 0.5) to each *m* and *n* before model selection using Bayesian information criterion. After the best *N* is selected, we use the original value of *m* and *n* (without the random noise) to train the GMM. The label of each read was predicted using the trained model. To make sure the subsequent SNP calling is accurate, a read is discarded if it is not within 95% equi-probability surface of a Gaussian model. A sample failed QC if only one allele was detected (*N* = 1) or one of the alleles had less than 50 reads.

### SNP/indel detection

To reduce the computational time, the reads of each allele were down-sampled to 200× coverage and then aligned to the human reference genome GRCh38 using minimap2^27^ with the parameter for nanopore reads (-x map-ont). The SAM file was converted to BAM file and sorted by SAMtools.^28^ SNP/indel calling was performed using longshot^29^ with default parameters. Homozygous calls of each allele were considered accurate and were used in downstream analysis.

### Generation of consensus sequence and structural variant detection

Reads of each allele were assembled by Canu (version: 2.0),^30^ and a consensus sequence was generated. The consensus sequence was aligned to the reference genome GRCh38 using minimap2^27^ with the parameter for assembly contigs (-x asm20). Structural variants (SVs) were called directly from the alignment using a custom pipeline.

### Analysis of gain and loss of PAMs mediated by SNPs

An SNP is considered to mediate the gain of a PAM if the alternative allele contains a high-efficiency PAM and the reference allele does not contain any high-efficiency PAM or low-efficiency PAM of the same CRISPR enzyme. Conversely, an SNP is considered to mediate the loss of a PAM if the reference allele contains a high-efficiency PAM and the alternative allele does not contain any high-efficiency PAM or low-efficiency PAM. TTTN is a high-efficiency PAM of AsCpf1. Both NGG and NAG are considered high-efficiency PAMs of the wild-type SpCas9, although the recognition for NAG is less efficient than NGG. For other Cas9 enzymes of which the post-selection PAM depletion values (PPVDs) are measured,^31^ a PAM is considered as a high-efficiency PAM if the PPVD is less than 0.2 (5-fold depletion). The list of high-efficiency and low-efficiency PAMs analyzed in this study is shown in Table S5.

### Sanger sequencing to validate SNP16 (rs3856973)

A 526-bp region flanking SNP16 (rs3856973, chr4:3078446G>A on GRCh38) was amplified by a nested PCR as we were not able to design a specific primer that directly amplifies this region. In the first round of the nested PCR, we used the primers of amplicon-2 (Table S2), which amplified an 8.8-kb region. The PCR condition was described in the above section. One microliter of the PCR product was used as the input of the second round of PCR. In the second round, the primers were 5′-TTGGGAGGGTCCTCACAGTA-3′ (forward) and 5′-GAGGTTGCAGTGAGCCAAGA-3′ (backward). The PCR conditions were: (1) 95°C for 3 min; (2) 98°C for 10 s; (3) 68°C for 45 s (30 cycles). Sanger sequencing was performed using the forward primer of the second-round PCR.

### TaqMan SNP genotyping assay to validate SNP16

The TaqMan Genotyping Master Mix (cat. no. 4,371,353) and the probes for SNP16 (assay ID: C__27529960_10) were ordered from Thermo Fisher Scientific. A 5-μL PCR reaction system was used. Real-time PCR and data analysis were performed following the manufacturer’s instructions.

### Cell culture and transfection

Human embryonic kidney (HEK293) cells (obtained from CHOP Research Vector Core stock) were maintained in DMEM medium containing 10% fetal bovine serum, 1% L-glutamine, and 1% penicillin/streptomycin at 37°C with 5% CO_2_. Cells were cultured in 24-well plates and transfected at 80%–90% confluence using Lipofectamine 2000 transfection reagent, according to the manufacturer’s protocol. After DNA transfection, cells expressing SaCas9 and sgRNA sequences were enriched by puromycin selection (3 μM) for 24 h, and subsequently expanded for genomic DNA and RNA extraction. Human HD fibroblasts (obtained from the Coriell Institute for Medical Research cell repository) were maintained on DMEM mediom supplemented with 10% fetal bovine serum, 1% MEM non-essential amino acids, 1% penicillin/streptomycin and 1% L-glutamine at 37°C with 5% CO_2_. DNA transfection was done by electroporation using an Invitrogen Neon transfection reagent using the electroporation conditions (ND33392: 1,450 V, 20 ms, 2 pulses), following the guidelines provided by the manufacturer. Fibroblasts were selected with puromycin (2 μM) for 24 h and subsequently expanded for genomic DNA extraction. Cells were not authenticated or tested for *Mycoplasma* by the investigators since they previously passed the quality controls of CHOP Research Vector Core and the Coriell Institute for Medical Research cell repository. None of the cells used in the study were listed in the ICLAC database of commonly misidentified cell lines.

### sgRNA and Cas9 plasmid construction

The plasmid pX330 containing the SpCas9 and sgRNA expression cassettes used in our previous study^20^ was used as a template to clone the SaCas9 cDNA and sgRNA sequences. To determine transfection efficacy and for selecting positively transfected cells, a CMV reporter cassette expressing eGFP/P2A/Puromicin fusion protein was cloned downstream of the SaCas9 expression cassette. For all sgRNAs, the guide complementary sequences were cloned using a single cloning step with a pair of partially complementary oligonucleotides. The oligo pairs encoding the genomic complementary guide sequences were annealed and ligated into the BbsI cloning site upstream and in frame with the invariant scaffold of the sgRNA sequence. The gRNA sequences for targeting SNP1, SNP16, and HDi3 are: GCCCCGCTCCAGGCGTCGGCG (SNP1), GATAGGGAAATGTCAGGGTTAA (SNP16), and GTGCTTTTAGGACGCCTCGGC (HDi3).

### RNA extraction and qRT-PCR

Total RNA was extracted using Trizol (Life Technologies) according to the manufacturer’s protocol, with the exception of 1 μL of Glycoblue (Life Technologies) in addition to the aqueous phase on the isopropanol precipitation step and a single wash with cold 70% ethanol. RNA samples were quantified by spectrophotometry and subsequently cDNAs generated from 1 μg of total RNA with random hexamers (TaqMan RT reagents, Applied Biosystems). To determine human HTT expression levels in HEK293 cells, we used TaqMan probes for human HTT and glyceraldehyde 3-phosphate dehydrogenase mRNAs obtained from Applied Biosystems. Relative HTT gene expression was determined using the ddCt method.

### Semiquantitative PCR for assessment of allele-specific editing

Allele-specific editing was assessed by semiquantitative PCR amplification of the CAG repeat within *HTT* exon-1. Genomic DNA (gDNA) was extracted from cultured HD fibroblasts using a QiaAMP DNA mini kit (QIAGEN) according to manufacturer’s instructions. The gDNA was quantified by fluorometry (Qubit) and then diluted to the same concentration (5 ng/μL). We used BIOLASE DNA polymerase (Bioline) to amplify the input DNA templates. As the CAG repeat and its flanking region has a high GC content (72.4%), we added betaine to the PCR reaction system to enhance amplification. The 50-μL PCR reaction system contains 8 μL gDNA (5 ng/μL), 5 μL 10× NH_4_ buffer, 2 μL dNTP mixture (2.5 mM each), 1.5 μL MgCl_2_ (50 mM), 1 μL BIOLASE DNA polymerase, 12 μL betaine (5 M), 1 μL primer mixture (10 μM each), and 19.5 μL ddH_2_O. The PCR thermal cycling program was 95°C for 2 min, followed by 25 cycles of 95°C for 15 s, 57°C for 15 s, 72°C for 45 s, and a final extension at 72°C for 5 min. The PCR products were separated by electrophoresis through a 1.5% agarose gel stained with ethidium bromide. The gel bands were quantified using the Image Lab software (Bio-Rad).

### Statistical analysis

Statistical analyses were performed using GraphPad Prism v.7 software. Outlier samples were detected using the Grubb’s test (a = 0.05). Normal distribution of the samples was determined by using the D’Agostino and Pearson normality test. Data were analyzed using one-way ANOVA followed by Bonferroni’s post hoc. Statistical significance was considered with p < 0.05. All results are shown as the mean ± SEM.

## Results

### Sequencing and data analysis workflow overview

The workflow of this study is shown in Figure 1. We used barcoded long-range PCR to amplify the target regions. The barcoded sequences were custom-designed such that: (1) there were neither tandem repeat sequences nor sequences similar to the human genome; (2) the pairwise sequence similarity between each two barcodes was minimized; (3) the GC content is between 40% and 60% (see material and methods; Figure S1). For both HD cohorts, we amplified the same target region (GRCh38, chr4:3069608-3079972), which begins 5,268 bp upstream of the CAG repeat and ends 5,096 bp downstream (Figure 1F; see material and methods for details). We used a combinatorial barcoding strategy, which adds different barcodes 5′ of both forward and backward primers (Tables S1–S3). The barcoded amplicons were pooled, size selected, and then sequenced in a MinION flow cell using the Oxford Nanopore GridION sequencer.

The sequencing data were de-multiplexed with NanoBinner, which was originally developed for this work. Given the moderately high error rate and the flexibility of alignment for long reads, there may be random barcode matches inside the amplicon sequence. To avoid potential random matching, NanoBinner compares the barcode sequence as well as the 256-bp amplicon sequence immediately next to the barcode. The 256-bp amplicon sequence acts as an anchor such that the matching of barcodes is at the correct position. NanoBinner also uses a new algorithm to assign barcodes, which is described in the material and methods section.

After de-multiplexing, CAG repeat sizes are detected by NanoRepeat, a novel repeat detection tool originally developed for this study. Through use of a novel algorithm, it jointly quantifies two adjacent tandem repeats, phases the reads using repeat sizes from both repeats, and reports haplotypes. In exon-1 of *HTT*, there is a CCG repeat immediately 3′ of the CAG repeat, with (CCG)_7_ and (CCG)_10_ predominant although there are other variants.^32^, ^33^, ^34^ The joint quantification of the CAG and CCG repeat has two steps. First, NanoRepeat performs a quick analysis of each repeat and independently determines the lower bound and upper bound of the repeat size. Next, NanoRepeat generates a batch of amplicon sequences with *m* (*L*_*1*_ ≤ *m* ≤ *U*_*1*_) CAG repeat units and *n* (*L*_*2*_ ≤ *n* ≤ *U*_*2*_) CCG repeat units, where *L*_*1*_, *L*_*2*_ are the lower bounds of the two repeats and *U*_*1*_, *U*_*2*_ are the upper bounds of the two repeats, respectively (Figure 1C). Each read is aligned to this batch of amplicon sequences using minimap2.^27^ The *m* and *n* that maximize the alignment score are the estimated CAG and CCG repeat sizes of the read (see material and methods for details). After the joint quantification, NanoRepeat uses the GMM to group the reads to alleles. Outlier reads are removed so that the subsequent SNP calling is accurate. At least 50 reads per allele are required for SNP calling. SNPs/Indels were detected using longshot.^29^

### Assessment of computational methods

**Figure 2:**
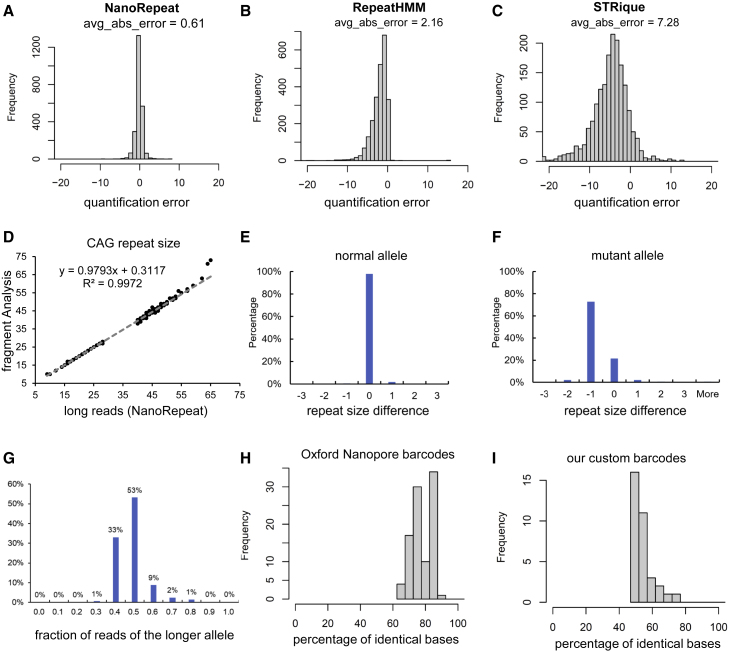
Assessment of computational methods (A–C) Benchmarking repeat quantification on 2370 STR regions in the CHM13 genome. The average absolute error (avg_abs_error) of each method is shown. (D) Scatterplot showing the CAG repeat size quantified by NanoRepeat and PCR-based fragment analysis. (E and F) Repeat size difference between NanoRepeat’s results and the PCR-based fragment analysis. (G) Distribution of the fraction of reads of the longer allele. (H and I) Histogram showing the percentage of identical bases between the barcode sequence and the best aligned sequence in the human reference genome GRCh38. Sequences of 96 Oxford Nanopore barcodes were obtained from the online documentation of the PCR Barcoding Expansion Pack.

We first tested the accuracy of repeat quantification in whole-genome sequencing data. We used the Oxford Nanopore dataset of the CHM13 genome.^35^ The CHM13 cell line has near-complete homozygosity, with only a few exceptions. The telomere-to-telomere consortium has finished the *de novo* assembly of CHM13 primarily based on PacBio HiFi reads, but supplemented with data from other sequencing platforms. As the CHM13 v.1.1 assembly sequence is highly reliable, repeat counts from the assembly sequence can be considered as a truth set for method assessment. We benchmarked NanoRepeat along with two other widely used repeat detection tools, namely RepeatHMM^36^ and STRique.^37^ Repeat detection was performed on 2,370 short tandem repeat (STR) regions in the CHM13 genome. These are all STR regions that are >100 bp and not within a 500-bp flanking region of another STR. We removed adjacent STRs because many of the adjacent STRs have similar sequences and it is hard to tell if they need to be merged or not without manual examination. The length of the 2,370 STR regions range from 100 to 2,374 bp. Their coordinates are shown in Table S10. The evaluation results are shown in Figures 2A–2C. NanoRepeat has much smaller quantification error compared with RepeatHMM and STRique. In addition, RepeatHMM and STRique have a systematic quantification bias which leads to underestimation of the repeat size.

Next, we assessed repeat detection in our HD samples. The CHDI Foundation provided the repeat sizes quantified earlier by PCR-based fragment analysis. We compared the repeat size quantified by NanoRepeat and the data provided by the CHDI Foundation. As shown in Figure 2D, NanoRepeat’s quantifications based on the long reads are highly consistent with the results of fragment analysis provided earlier (R^2^ = 0.9972). For normal alleles, the two methods were identical for approximately 98% of samples (Figure 2E). For expanded alleles, repeat size differences between the two methods were within one repeat unit for more than 95% samples (Figure 2F). There is very little bias between the normal and the expanded alleles (Figure 2G).

We also compared our custom-designed barcodes with the 96 barcodes provided by ONT. We observed that 35 of the 96 barcodes in ONT’s PCR Barcoding Expansion Pack can be aligned to the human reference genome GRCh38 with more than 80% identical bases (Figure 2H). For example, BC71 is 24 bp and can be aligned to chr18:46568247-46568269 (GRCh38) with 22 bp matched. To avoid potential non-specific binding in the PCR reaction, we designed our own barcodes. As shown in Figure 2I, none of our custom barcodes has more than 80% identical bases in the human reference genome GRCh38.

### QC summary of the amplicon sequencing experiments

In total, 318 samples from the French HD consortium and 664 samples from the CHDI Foundation passed QC and had sufficient sequencing data to make reliable variant calls. To ensure accurate SNP detection, we require a stringent QC criterion: at least 50× coverage for each allele. The QC summary of the amplicon sequencing experiments is shown in Table 1. The French cohort was amplified twice with different primers. Amplicon-1 (5.9 kb) mainly covers the upstream region of exon-1 while amplicon-2 (8.8 kb) mainly covers the downstream region. The two regions have some overlap and both cover the CAG repeat region. Therefore, a full-length haplotype can be assembled from phased SNPs of the two amplicons. For amplicon-1, 93% (370/396) of the samples passed QC while 90% (355/396) of the samples passed QC for amplicon-2. Eighty-eight percent (348/396) of samples passed QC for both amplicons and were used to assemble the full-length haplotype.

**Table 1:** Quality control summary of the amplicon sequencing experiments

Cohort	French	French	CHDI
Total no. of samples	396	396	960
Amplicon	amplicon-1	amplicon-2	amplicon-3
Amplicon length (bp)	5,910	8,854	10,365
No. of pooled samples per flow cell	100	100	480
QC-passed samples	370	355	708
Pass rate (%)	93	90	74

The CHDI cohort was amplified using the forward primer of amplicon-1 and backward primer of amplicon-2. This amplicon is referred to as amplicon-3 (10.3 kb; Figure 1F). A total of 74% (708/960) samples passed QC. This rate is lower than that of the French cohort, probably due to (1) the length of amplicon-3 is longer than amplicon-1 and amplicon-2, thus amplicon-3 is more difficult to amplify and requires higher DNA integrity; (2) some samples are of low concentration. Therefore, we used the French cohort as the main cohort for analysis and validate our conclusions on the CHDI cohort.

### CAG and CCG repeat sizes detected from HD samples

**Figure 3:**
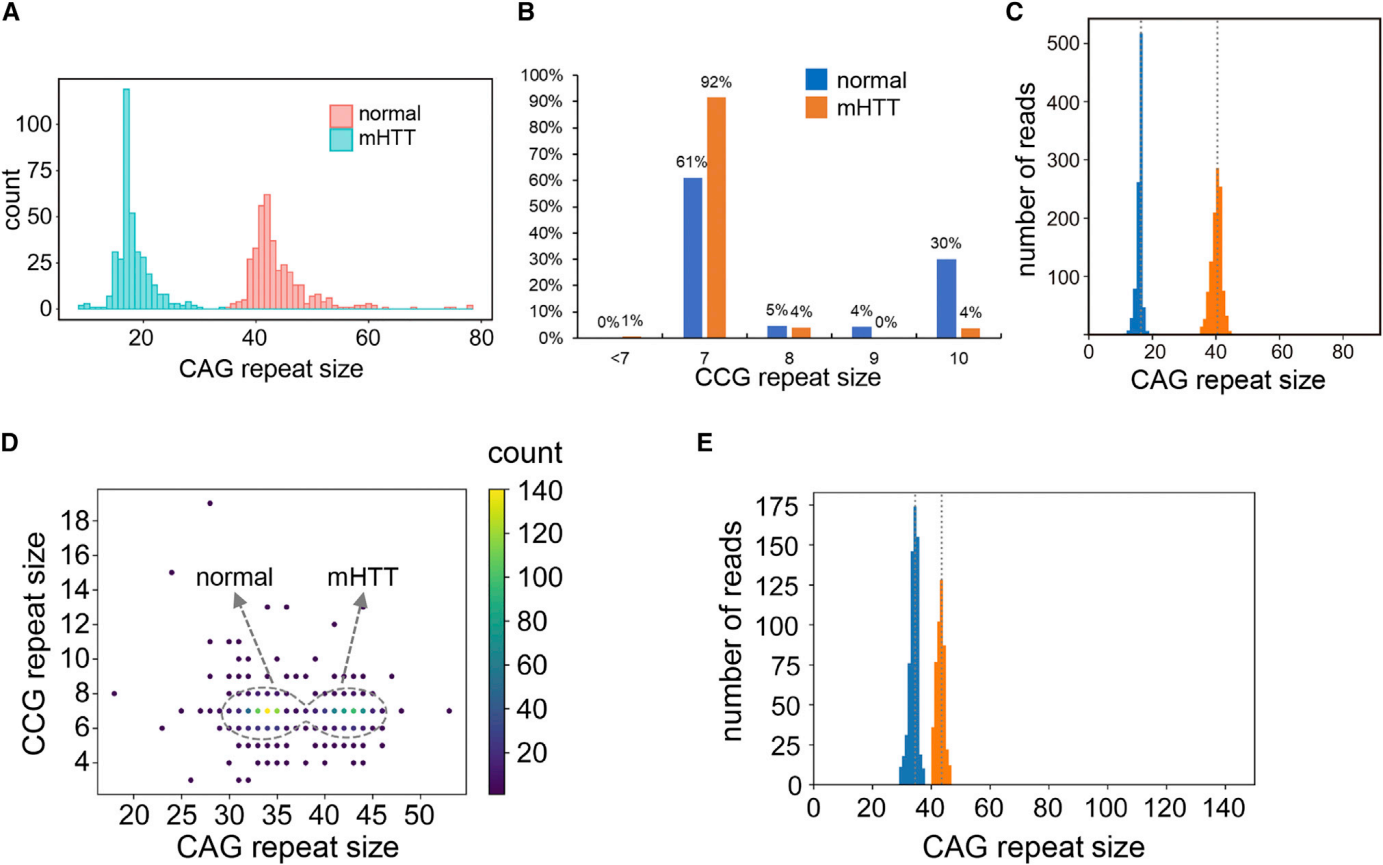
CAG and CCG repeats detected from HD samples (A) Distribution of CAG repeat size of the French cohort. (B) The normal and mHTT allele showed distinct distribution in CCG repeat sizes. (C) CAG repeat size distribution of a typical HD sample. The reads derived from the normal allele (blue) and the *mHTT* allele (orange) are well separated. The estimated repeat sizes are marked by vertical gray lines. (D) Scatterplot of a sample with a disease-causing allele (CAG repeat size = 43) and an intermediate allele (CAG repeat size = 34). The dashed gray circle is an equi-probability surface of the fitted Gaussian models where the probability outside the surface is less than 5%. (E) Distribution of CAG repeat size of the same sample shown in (D). Reads that are not confidently classified are removed. The two alleles are well separated (blue, normal allele; orange, *mHTT* allele). The estimated repeat sizes are marked by vertical gray lines.

The distribution of CAG repeat size is shown in Figure 3A (French cohort) and Figure S2 (CHDI cohort). Alleles with ≤35 CAG repeat units are denoted as normal alleles, and those with ≥36 CAG repeat units denoted as expanded alleles. Most normal alleles are less than 26 repeats; repeat sizes between 27 and 35 were rare, consistent with previous studies.^24^ CCG repeat size distribution were different among normal and *mHTT* chromosomes (Figure 3B). For normal, there were two predominant alleles: (CCG)_7_ and (CCG)_10_. In *mHTT* chromosomes, (CCG)_7_ predominates (∼92%).

Since there was often a large difference (>20) in CAG repeat size between the normal and *mHTT* chromosomes, it was easy to separate the reads derived from normal and *mHTT* alleles for most samples (Figure 3C). One sample had CAG repeat sizes of 34 (intermediate) and 43 (expanded), which is the smallest CAG repeat size difference in the French HD cohort; both alleles have the same CCG repeat size. With the Gaussian mixture models, we were able to compute the probabilities and remove the reads that were not confidently classified so that the two alleles were well separated (Figures 3D and 3E).

### SNPs with high allele frequency in HD samples

After the reads were phased according to the CAG and CCG repeats, SNP detection was performed for each allele (see material and methods section), generating a list of haplotyped SNPs of 10.3 kb. The complete list of the SNPs is shown in Tables S4, S13, and S14. A total of 110 SNPs were identified from the two HD cohorts; 56 SNPs are in the upstream HTT gene region; one SNPs is in 5′ UTR; 7 SNPs are in the exon-1 coding region, and 46 SNPs are in intron-1. Eight SNPs are novel and have not been found in dbSNP, the Genome Aggregation Database (gnomAD), or the 1000 Genomes Project.^38^ We validated the eight novel SNPs by examining the alignments using the Integrative Genomics Viewer (IGV^39^), we acknowledge that such novel SNPs could be still due to errors in long-range PCR and subsequent sequencing. The IGV screenshots are displayed in Figures S5–S12.

**Figure 4:**
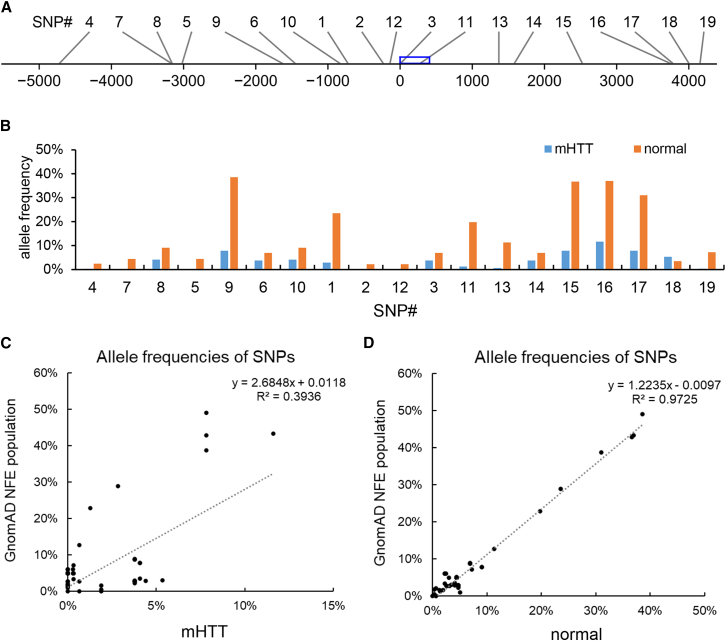
SNPs detected from the HD samples (A) Positions of 19 common SNPs (relative to the first base of the *HTT* exon-1). The blue box indicates the position of exon-1. (B) AFs of 19 common SNPs identified in the HD cohort. The details of the SNPs are shown in Table 2. (C) Scatterplot showing the AFs of SNPs in HD *mHTT* alleles and the gnomAD database (NFE population). (D) Scatterplot showing the AFs of SNPs in normal alleles and the gnomAD database (NFE population). (B–D) are based on the data of the French HD cohort.

We first analyzed the SNPs identified from the French cohort. Among those identified, 19 are common with allele frequencies (AFs) of ≥5% in the normal or *mHTT* chromosomes, or the gnomAD database (non-Finnish European [NFE] population). The positions of the 19 SNPs are shown in Figure 4A. Notably, their AFs in normal and *mHTT* chromosomes are dramatically different (Figure 4B; Table 2). To verify SNP detection, the AFs of all identified SNPs were compared with their AFs in the gnomAD database. The AFs of SNPs in the normal chromosomes are highly correlated (R^2^ = 0.9725) with their AFs in NFE population in the gnomAD database. However, the correlation between the AFs of SNPs in the *mHTT* chromosomes and those in the gnomAD database are low (R^2^ = 0.3936) (Figures 4C and 4D). This indicates unique haplotypes for *mHTT* chromosomes, which is consistent with previous findings that CAG expansion events are associated with specific haplogroups.^24^ Examination of the AFs of SNPs identified from the CHID cohort validated our findings (Figure S3).

**Table 2:** Common SNPs identified in the HD cohort

SNP ID	Position in Chr4	Accession no.	Ref allele	Alt allele	AF expanded (%)	AF normal (%)	AF gnomAD (NFE) (%)	Enzyme	Ref motif	Alt motif	Effect on PAM (gain/loss)	Strand
SNP4	3069952	rs35631490	C	G	0.3	2.6	6.0	SpCas9	TG[G]	TG[C]	loss	negative
SNP7	3071526	rs77384845	C	T	0.0	3.7	5. 1	SpCas9 VQR	T[G]AC	T[A]AC	loss	negative
SNP8	3071527	rs10011412	A	G	4.3	7.5	7.8	—	—	—	—	—
SNP5	3071658	rs61792464	G	C	0.3	3.7	5.1	SpCas9	GG[C]	GG[G]	gain	negative
								SpCas9	AA[G]	AA[C]	loss	positive
SNP9	3073068	rs762855	A	G	6.3	32.5	49.0	—	—	—	—	—
SNP6	3073238	rs9996199	C	G	4.3	6.9	8.8	SpCas9	TA[C]	TA[G]	gain	positive
								SpCas9	CA[G]	CA[C]	loss	negative
								SpCas9 VQR	A[C]TG	A[G]TG	gain	positive
SNP10	3073861	rs28431418	T	C	4.6	8.1	7.9	Sau Cas9	GTGAG[T]	GTGAG[C]	loss	positive
SNP1	3073964	rs2857935	G	C	2.6	22.7	29.0	SpCas9	GG[G]	GG[C]	loss	positive
								SpCas9 VQR	GGG[G]	GGG[C]	loss	positive
								SpCas9 VQR	G[G]AT	G[C]AT	loss	positive
								Sau Cas9	GGG[G]AT	GGG[C]AT	loss	positive
SNP2	3074454	rs13122415	C	G	0.3	2.6	6.0	SpCas9	AG[G]	AG[C]	loss	negative
								SpCas9	G[G]G	G[C]G	loss	negative
								SpCas9 VQR	G[G]GG	G[C]GG	loss	negative
SNP11	3074945	rs76533208	A	G	1.7	24.4	22.8	SpCas9 VRER	GG[T]G	GG[C]G	gain	negative
SNP12	3074539	rs13132932	A	G	0.00	2.6	6.0	SpCas9 VQR	GGC[A]	GGC[G]	gain	positive
								SpCas9 VRER	GGC[A]	GGC[G]	gain	positive
								SpCas9 VQR	C[A]GG	C[G]GG	gain	positive
SNP3	3074678	rs13102260	G	A	4.3	6.90	9.0	SpCas9	TG[G]	TG[A]	loss	positive
								SpCas9 VQR	G[G]GG	G[A]GG	loss	positive
								SpCas9 EQR	TG[G]G	TG[A]G	gain	positive
SNP13	3076049	rs73191179	G	A	0.9	10.9	12.7	SpCas9 VQR	C[G]GG	C[A]GG	loss	positive
SNP14	3076258	rs28656215	T	C	4.3	6.90	8.8	—	—	—	—	—
SNP15	3077210	rs3905238	A	G	8.1	35.3	42.9	SpCas9 VQR	TGC[A]	TGC[G]	gain	positive
								SpCas9 EQR	TGC[A]	TGC[G]	gain	positive
SNP16	3078446	rs3856973	G	A	12.1	35.1	43.3	SpCas9 VQR	C[G]AG	C[A]AG	loss	positive
								SpCas9 EQR	C[G]AG	C[A]AG	loss	positive
								Sau Cas9	TC[G]AGT	TC[A]AGT	loss	positive
SNP17	3078472	rs4498089	A	G	8.1	29.6	38.7	SpCas9	AA[A]	AA[G]	gain	positive
								SpCas9 VQR	A[A]AA	A[G]AA	gain	positive
								AsCpf1	TT[T]T	TT[C]T	loss	negative
SNP18	3078688	rs148125464	C	T	5.2	2.9	3.1	SpCas9	AG[G]	AG[A]	loss	negative
								SpCas9 VQR	AG[G]C	AG[A]C	gain	negative
SNP19	3078835	rs57666989	C	T	0.9	7.8	7.2	SpCas9 VQR	GGC[G]	GGC[A]	loss	negative
								SpCas9 VRER	GGC[G]	GGC[A]	loss	negative
								SpCas9 VQR	C[G]TG	C[A]TG	loss	negative

Allele frequencies and effect on PAM are listed.

### Identification of SNPs for allele-specific genome editing

We analyzed the gain and loss of PAMs mediated by the 19 common SNPs as they comprise the majority of diversity in HD individuals and the general population, yielding putative targets for allele-specific genome editing. In addition to the canonical PAMs (NGG and NAG) recognized by SpCas9, we analyzed PAMs for five other native or engineered CRISPR enzymes, namely SaCas9, SpCas9_VQR, SpCas9_EQR, SpCas9_VRER, and AsCpf1. SaCas9 is a Cas9 ortholog from *Staphylococcus aureus*.^40^ SpCas9_VQR, SpCas9_EQR and SpCas9_VRER are engineered SpCas9 variants from *Streptococcus pyogenes*. AsCpf1 is a class 2 CRISPR enzyme from *Acidaminococcus*, which is highly specific for the AT-rich motif (TTTN). Only highly effective PAMs recognized by these enzymes were analyzed (Table S5). An SNP can mediate the loss, gain, or loss/gain of PAMs and may induce or destroy PAMs for multiple enzymes. Two types of SNPs can mediate the deletion of the *mHTT*: (1) SNPs causing loss of a PAM in the normal chromosome; and (2) SNPs causing gain of a PAM in the *mHTT* chromosome. The effect of the 19 SNPs on different PAMs, and their AF, is shown in Table 2.

Among them, SNPs 1–6 are identical to the six SNPs previously identified and tested.^20^ All have much higher AFs in the normal chromosomes than in the *mHTT* chromosomes (Table 2; Figure 4B). For example, the AF of SNP1 in the normal chromosomes is 23.51%, which is 8.3 times of the AF in the *mHTT* chromosomes (2.82%). All six SNPs cause PAM loss. Thus, the bias in AF (higher abundances on the normal chromosomes) allows HD individuals to benefit from this genome editing strategy. This is consistent with our previous observations that SNP1 mediated deletion of the expanded HTT in 9 of 11 cell lines (see Table S4 of Monteys et al.^20^).

Sixteen of the 19 SNPs cause the loss or gain of at least one PAM. In addition to SNPs1-6, SNP16 and SNP17 mediate PAM loss in the normal allele, with an AF > 30% in the normal chromosomes. The AFs of SNP16 in normal and *mHTT* chromosomes are 36.99% and 11.6%, respectively. The alternative allele of SNP16 disrupts the PAM of SpCas9_VQR and SpCas9_EQR (ref: C[G]AG; alt: C[A]AG). Both SpCas9_VQR and SpCas9_EQR recognize the PAM of NGAG, but SpCas9_VQR also recognizes the motif of NAAG, at lower efficiency.^31^ SNP16 can also be targeted by SaCas9 (ref: TC[G]AGT; alt: TC[A]AGT). SNP17 resides in a ploy-T region and exists in 31.03% of normal chromosomes assessed, and in 7.84% of *mHTT* chromosomes. SNP17 disrupts the PAM of AsCpf1 (ref: TT[T]T; alt: TT[C]T), which has a strong selectivity for the TTTN PAMs and thus SNP17 is AsCpf1 specific.

The specificity of the guide RNA sequence is critical for preventing off-target cleavage events. For the Cas9 enzyme and its variants, the guide sequence is on the 5′ side of the PAM. But for the Cpf1 enzymes, the guide sequence is on the 3′ side of the PAM. We used the Benchling website to design guide RNAs and predict their specificity. The guide RNA sequence for SNP16 is very specific with an off-target score of 98.4 (score ranging between 0 and 100, the higher the better). The guide RNA sequence (TAAAAATAAAAATAAGTTAACAC) for SNP17 is not specific, with an off-target score of 43.3. This sequence contains a poly(A) and may have multiple copies in the human genome. Therefore, SNP16 is a strong candidate with high AF and specificity.

We validated SNP16 genotyping from long reads using two traditional methods. First, we designed Sanger sequencing primers for SNP16 and randomly sequenced 20 samples from plate-1 of the French cohort. The Sanger sequencing results were completely consistent with the SNP genotypes called from the nanopore long reads. We also used TaqMan real-time PCR assay to genotype three 96-well plates (288 samples) of the CHDI cohort. The TaqMan assay results were also completely consistent with the SNPs called from the nanopore long reads. This indicates that our SNP calls made from the nanopore long reads are of high accuracy.

### Experimental validation of the allele-specific cleavage mediated by SNP16

**Figure 5:**
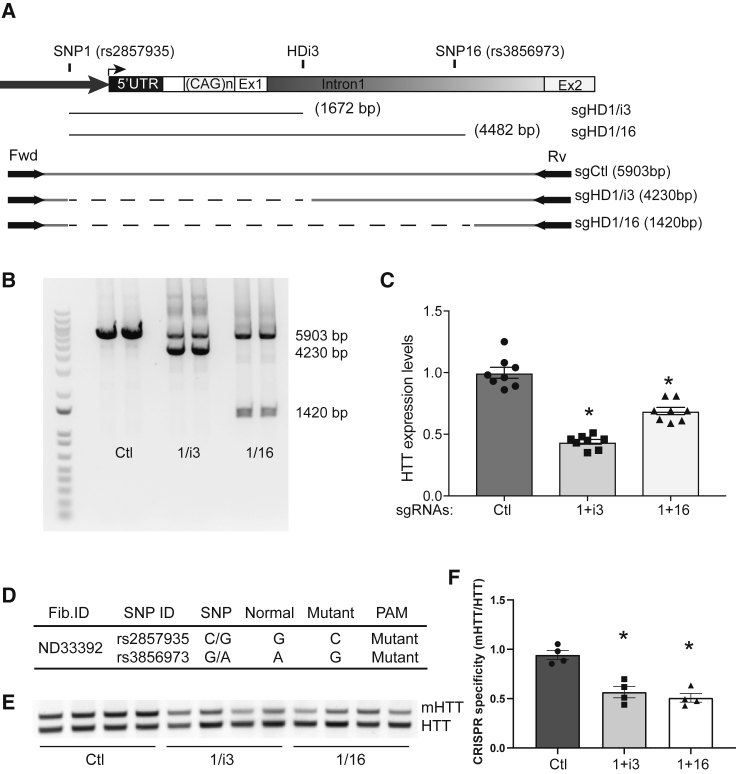
Allele-specific cleavage mediated by SNP1 and SNP16 (A) Cartoon depicting the relative position of SNP1- and SNP16-dependent PAMs flanking *HTT* exon-1 and one common PAM within *HTT* intron-1 (HDi3). PCR primer positions and estimated sizes of the targeted deleted sequences are indicated. 1, i3, and 16 are sgRNAs targeting PAMs at SNP1, HDi3, and SNP16, respectively. (B) A genomic PCR showing *HTT* exon-1-targeted deletion mediated by SNP1- and SNP16-dependent PAMs. (C) qRT-PCR analysis of *HTT* mRNA levels in HEK293 cells transfected with CRISPR enzymes targeting SNP1 and SNP16 (n = 8). (E) The haplotype of the ND33392 fibroblast cell line and the corresponding PAMs. (F) A semiquantitative PCR reaction showing the reduction of the *mHTT* allele in a fibroblast cell line (ND33392) transfected with SaCas9 targeting SNP1 and SNP16. (C) and (F) The results are mean ± SEM relative to the control group. ∗p < 0.05, one-way ANOVA followed by Bonferroni’s post-hoc.

Our screen identified SNP16 (rs3856973) as a novel prevalent SNP within *HTT* intron-1, which could be used alone or together with SNP1 (rs2857935) to edit and terminate *mHTT* expression. We designed sgRNA sequences targeting SNP16 (sgHD16) and SNP1 (sgHD1) to mediate SaCas9 editing of the *mHTT* exon-1. The sgHDi3 (targeting intron-1) and sgCtl guides used in our previous study^20^ were modified to complex with SaCas9. We tested the editing efficacy of three pairs: sgHD1/16 (targeting SNP1 and SNP16), sgHD1/i3 (previously tested, positive control), and sgCtrl (negative control) (Figure 5A). HEK293 cells, which are homozygous for both SNP1 and SNP16, were transfected with sgRNA/SaCas9 expression plasmids and genomic deletions were assessed. DNA products of the anticipated size were amplified in all sgRNA/SaCas9 pairs tested. The *HTT* genomic locus remained intact on cells co-expressing SaCas9 and sgCtl (negative control group), whereas a band resulting from *HTT* exon-1 deletion was observed on cells transfected with the sgHD1/i3 SaCas9 or the sgHD1/16 SaCas9 cassettes (Figure 5B). Notably, the intensity of the amplified DNA bands indicates that the editing efficacy of sgHD1/i3 was higher than the sgHD1/16 pair. HTT mRNA levels were reduced in cells following editing, as determined by qPCR (Figure 5C). Reduction of HTT mRNA levels was greater in cells expressing sgHD1/i3 than the sgHD1/16 pairs, mirroring what was observed by PCR of genomic DNA.

Next, we tested if the sgHD1/16 pair can be used for expanded allele-specific targeting. The ND33392 fibroblast line contains heterozygous SNP1 and SNP16 with the SNP-dependent PAMs on the *mHTT* allele (Figure 5D). PCR amplification of genomic DNA using primer pairs binding within the *mHTT* exon-1 sequence showed targeted cleavage of the *mHTT* allele in cells electroporated with plasmids expressing sgHD1/i3/SaCas9 and sgHD1/16/SaCas9 relative to those electroporated with the control sgCtl/SaCas9 complex (Figures 5E and 5F).

### Analysis of haplotypes of the HD samples

To better understand the percentage of HD individuals who could benefit from our editing approach, the haplotypes of normal and *mHTT* chromosomes of the HD individuals as well as those in the 1000 Genomes Project phase 3 dataset (denoted as 1KG dataset hereafter) were analyzed. The 1KG dataset provides phased SNP/indel calls of 2,504 individuals, of whom 404 individuals are from the NFE population. In the following haplotype analysis, we used all SNPs listed in Table 2 except SNP11, as it resides in the CAG-CCG repeat region and was not genotyped in the 1KG dataset.

**Figure 6:**
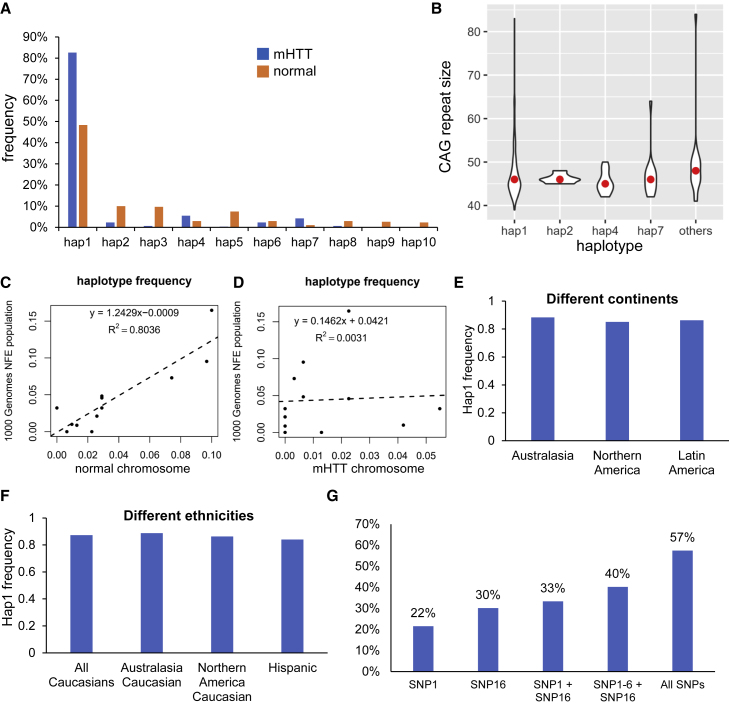
Haplotype analysis of HD individuals (A) Frequencies of top 10 haplotypes of HD samples. (B) Violin plot showing the distribution of repeat sizes in different haplotypes of the *mHTT* chromosomes. The red dots indicate the median of the repeat size. (C) Scatterplot showing haplotype frequencies of the normal chromosomes and 1000 Genomes individuals (phase 3, non-Finnish European population). (D) Scatterplot showing haplotype frequencies of the *mHTT* chromosomes and 1000 Genomes individuals (phase 3, non-Finnish European population). (E and F) Frequencies of Hap1 in *mHTT* chromosomes in HD samples from different continents and ethnicities. (G) Percentage of HD individuals who can be edited, based on the haplotype analysis.

In total, we observed 37 different haplotypes in the French HD cohort (including normal and *mHTT* chromosomes) and individuals of NFE population in the phase 3 dataset. The SNPs carried by each haplotype are shown in Table S6. The frequencies of the top 10 most abundant haplotypes are shown in Figure 6A. Hap1 (no SNP across the region) is the predominant haplotype (82.58%) in the *mHTT* chromosomes. It is also the most abundant haplotype in normal chromosomes (frequency = 48.39%). The frequencies of minor haplotypes (haplotypes 2–37) are dramatically different between normal and *mHTT* chromosomes. The frequencies of the minor haplotypes in the normal chromosomes are highly correlated (R^2^ = 0.8036) with those in the 1KG dataset. In contrast, the haplotype frequencies in the *mHTT* chromosomes have no correlation (R^2^ = 0.0031) with those in the 1KG dataset (Figures 6C and 6D). To validate these discoveries, we examined the haplotypes of the CHDI cohort, which contains HD samples from multiple continents and ethnicities. Similar to the French cohort, Hap1 is the predominant haplotype (>80%) of the *mHTT* chromosomes, in samples from all continents and ethnic groups with more than 50 samples (Figures 6E and 6F). We also tested if there is an association between the expanded CAG repeats and haplotypes. The median CAG repeat size is between 45 and 48 in all groups (Figure 6B). We did not find a haplotype that is associated with specific repeat sizes.

Since the haplotypes of all HD individuals have been identified, we could estimate the percentage of people potentially amenable to our allele-specific genome editing strategy (Figure 6I); 22% of the HD individuals can be edited at SNP1 and 30.2% of the HD individuals can be edited at SNP16. The fraction of HD subjects who have SNP16 on both mutant and normal HTT, mutant HTT only, normal HTT only, and none are 4.9%, 7.2%, 30.2%, and 57.8%, respectively. A combination of SNPs1-6 and SNP16 can potentially edit 41% HD individuals. Up to 57% HD individuals can be edited if all SNPs are targeted. However, 43% HD individuals cannot be edited as 91% of them carry homozygous hap1 and there is no heterozygous SNP in the region covered by our sequencing data (5,073 bp upstream and 4,884 bp downstream of exon-1).

### Searching for potential editing sites for individuals with hap1 in both alleles

We tried additional strategies to find potential editing sites for individuals with hap1 in both alleles. First, we called indels and structural variants (SVs) from our sequencing data. However, all common Indels/SVs are located in tandem repeat regions where the guide RNAs are not specific. Next, we analyzed SNPs within a greater range than our earlier 10-kb limit using the 1KG dataset (NFE population). Since the 1KG dataset provides phased SNPs, we can extract the SNPs from chromosome 4 with different haplotypes. We found that, in hap1 chromosomes, there are fewer SNPs in the *HTT* gene (167 kb) compared with the intergenic region. (Figure S4). In regions upstream of the *HTT* gene, the nearest high-frequency SNP (AF > 20%) in hap1 chromosomes is 12.4 kb away from exon-1 (Table S7), which is outside of the range useful for efficient editing. In the region downstream of exon-1, the closest high-frequency SNP is 20.7 kb away (Table S8), similarly of limited utility for deleting exon-1. However, this SNP resides close to exon-3 and could be a candidate site to delete exon-3. We also found several high-frequency SNPs close to exon-6, exon-17, exon-23, and others. The *HTT* gene is a large gene with 67 exons. Theoretically, deletion of exon-3/exon-6/exon-23 would shift the reading frame and cause a premature stop codon disrupting huntingtin protein expression. However, if expanded exon-1 is left intact, alternative splicing events or RAN translation could still occur.^41^, ^42^, ^43^

## Discussion

In this study, we developed a targeted long-read sequencing approach to resolve a 10.4-kb genomic region flanking the CAG repeats in exon-1 of the *HTT* gene and applied this approach to two independent HD cohorts for the purpose of allele-specific editing for HD therapeutic development. We called genomic variants from the sequencing data and systematically analyzed potential gene-editing sites that could mediate allele-specific deletions of the *mHTT* allele. Our results showed that 22% of HD individuals can be edited by targeting SNP1, to which the guide RNA had been developed and tested earlier.^20^ In addition, we identified SNP16 as a novel candidate, which can target more people (30%) than SNP1 does in HD individuals of European ancestry. In proof-of-concept experiments, gRNAs targeting SNP16 could effectively edit *mHTT* cell lines. Overall, our haplotype analysis reveals that up to 57% HD individuals of European ancestry can be potentially targeted in an allele-specific manner by combinatorial editing.

Hap1 is the predominant haplotype in both normal chromosomes (48.39%) and *mHTT* chromosomes (82.58%). Due to the lack of diversity in the haplotypes, about 40% of HD individuals carry hap1 in both alleles and cannot be edited by targeting an SNP near exon-1. Unfortunately, further analyses using other strategies did not find high-frequency Indels or SVs that could mediate allele-specific editing; the nearest high-frequency SNP was at least 10 kb away. Despite this, deletion of other exons in an allele-specific manner is possible. Their deletion could induce an open reading frameshift causing a premature stop codon and loss of *mHTT* expression. However, toxicity from exon-1-derived transcripts would remain.^41^, ^42^, ^43^

Earlier work also analyzed SNPs and HD haplotypes.^23^^,^^44^^,^^45^ For this, SNP arrays were used to genotype common SNPs and focused on target sites for allele-specific knockdown by ASOs or RNAi. Thus the haplotypes were based on the *HTT* gene, most of which are distant from exon-1. Here, we used long-read sequencing. This has the advantage over SNP array genotyping and short-read sequencing in that it can perform repeat quantification, SNP detection, and haplotyping at the same time. With long reads, the haplotyping process is straightforward and does not require trio data. We were able to assemble the diploid genome sequence for each HD individual. However, we want to stress that, while the genetic details that we observe provide some potentially interesting genetic insights into how the various haplotypes might have arisen, we do not have evidence that this specific haplotype is responsible for the HTT expansion observed; instead, it is quite likely that the expansion arose on a specific haplotype, which is then overrepresented in the HD population across the continent. Cumulatively, our data provide a comprehensive analysis of allele-specific target sites for CRISPR-based gene editing, which relies on the ability of an SNP to provide a PAM site for targeted editing of the expanded allele. For effective editing, we focused on genomic regions within 5 kb of exon-1, because previous work showed that the distance between upstream and downstream guides influenced editing efficacy.^20^

Long reads have a higher per-base error rate than short reads. However, the sequencing error tends to be random and the consensus sequence of high-coverage long reads can be very accurate.^46^ We required at least 50× coverage per allele for analysis, and the vast majority of samples have more than 200× coverage per allele. In our results, the AFs of the SNPs in normal chromosomes are highly correlated with those in the gnomAD database, indicating that the SNP detection is correct. The haplotype frequencies in normal chromosomes are also highly correlated with those in the 1KG dataset. In addition, we validated the genotypes of SNP16 using Sanger sequencing and a TaqMan SNP genotyping assay, both of which generated results identical to the ONT long reads. Of note, ONT sequencing produces more Indel errors in homopolymer regions (e.g., the poly(A) sequence). Therefore, indel detection from ONT reads may be less accurate in homopolymer regions. As our study focuses on SNP detection, our data and are less affected by this limitation.

NanoBinner and NanoRepeat are novel computational tools developed for this work. NanoBinner is a demultiplexer for amplicon sequencing data. Existing tools, such as DeepBinner^47^ and qcat, only support the barcoding kits provided by ONT. NanoBinner is a general tool and can work with any user-provided barcodes. NanoRepeat is a tool for repeat detection from amplicon sequencing. It uses alignment-based quantification and can jointly quantify two adjacent tandem repeats, phase the reads, and report haplotypes. We evaluated NanoRepeat on both whole-genome sequencing data and amplicon sequencing data of our HD cohort. In the whole-genome data, NanoRepeat outperformed other repeat quantification tools and reduced the average quantification error by 3.5-fold. In our amplicon sequencing data of the CHDI cohort, NanoRepeat is highly consistent with PCR-based fragment analysis, which is commonly used in clinical labs for diagnostic purposes.

sgRNAs targeting SNPs1-6 were validated previously.^20^ In this study, we developed sgRNAs targeting SNP16 and tested it in cell lines. There was significant reduction of HTT levels in HEK293 cells and *mHTT* in ND33392 fibroblast cells. However, the efficacy of editing SNP1/16 (two SNPs) was not higher than editing SNP1/i3 (one SNP and one homozygous site), which may due to the longer distance between the SNP1/16 sgRNA-Cas9 complexes and sequence context at SNP16. Nonetheless, SNP16 is a promising candidate because it creates a PAM more frequently than SNP1 and editing at SNP16 would benefit HD individuals without SNP1.

In summary, we developed an experimental and computational workflow to resolve the SNP haplotypes near exon-1 of the *HTT* gene for allele specific editing. We applied this workflow to two HD cohorts and comprehensively analyzed potential sites for allele-specific deletion of *mHTT* for CRISPR-Cas systems. We also generated a detailed haplotype map for the region near HTT exon-1, which may be applied to other editing strategies and newly emerging editing enzymes. In addition, our workflow and novel computational tools can be applied to other repeat expansion disorders.

### Ethics statements

This study was approved by the Institutional Review Board at the CHOP Research Institute. HD participants from the French cohort were recruited at the Pitié Salpetrière Hospital in Paris, France. All participants gave written informed consent, and blood samples were collected in accordance with local French regulations (Paris Necker ethics committee approval [RBM 03–48] to A.D.). Genomic DNA samples from the CHDI cohort were generously provided by the participants in the Enroll-HD study and made available by the CHDI Foundation.

## Data Availability

Due to potential compromise of individual privacy, the raw sequencing data of the HD individuals generated during this study has not been deposited in a public repository but are available from the corresponding author on reasonable request and institutional data use agreement. The code (NanoBinner and NanoRepeat) generated during this study is available on GitHub. A tutorial for statistical phasing based on tagging SNPs (using data in this study) is also available on GitHub.

## Web resources

Benchling research platform, https://benchling.com

NanoBinner: https://github.com/WGLab/NanoBinner

NanoRepeat: https://github.com/WGLab/NanoRepeat

OMIM, https://www.omim.org

Qcat, https://github.com/nanoporetech/qcat

A tutorial for statistical phasing based on tagging SNPs, https://github.com/WGLab/HTT-SNP-Phasing

## Supplemental information

Document S1. Figures S1–S12 and Tables S1–S3, S5, S7–S9, and S12

Table S4. SNP frequencies in normal and mutant HTT The SNPs are annotated with frequencies in different databases including GnomAD v3.0, GnomAD v2.1.1, and 1000 Genomes Project Phase 3.

Table S6. SNPs carried by each haplotype The genome coordinates are based on GRCh38. ‘0’ indicates the reference allele and ‘1’ indicates the alternative allele.

Table S10. The list of filtered STR regions in CHM13 for evaluation of repeat quantification This list includes all STR regions that are > 100 bp and not within a 500 bp flanking region of another STR. We removed adjacent STRs because many of the adjacent STRs have similar sequences and it is hard to tell if they need to be merged or not without manual examination. Percent_match and percent_indel were calculated by Tandem Repeat Finder (TRF) v4.09.

Table S11. Detailed information of the CHDI cohort Race, sex, region, and CAG repeat size (measured by PCR-based Fragment Analysis) of each subject are shown. Subjects are deidentified. This information was provided by the CHDI foundation.

Table S13. Phased SNPs of each individual in the French cohort The genome coordinates are based on GRCh38.

Table S14. Phased SNPs of each individual in the CHDI cohort The genome coordinates are based on GRCh38.

Table S15. CAG and CCG repeat sizes for the French cohort The repeat sizes were quantified by NanoRepeat from Oxford Nanopore long reads.

Table S16. CAG and CCG repeat sizes for the CHDI cohort The repeat sizes were quantified by NanoRepeat from Oxford Nanopore long reads.

Document S2. Article plus supplemental information
