# Haplotyping SNPs for allele-specific gene editing of the expanded huntingtin allele using long-read sequencing

**DOI:** 10.1016/j.xhgg.2023.100212

**Published:** 2023-06-20

**Authors:** Li Fang, Alex Mas Monteys, Alexandra Dürr, Megan Keiser, Congsheng Cheng, Akhil Harapanahalli, Pedro Gonzalez-Alegre, Beverly L. Davidson, Kai Wang

## Main text

(Human Genetics and Genomics Advances *4*, 100146; January 12, 2023)

In the originally published version of this article, the data availability statement applies to the discovery cohort (French HD cohort) only. We changed this statement to: “The data of the French HD cohort is available from the corresponding author on reasonable request and institutional data use agreement. For queries to obtain biomaterials, raw and processed data of the CHDI cohort, please contact CHDI Foundation (info@chdifoundation.org).”

In the originally published version of this article, we included information from CHDI in Tables S11, S14, and S16 inadvertently. This is considered as Material-Related Information by CHDI. We removed the sample-related columns of Tables S11, S14, and S16 and added the following sentence “Sample-related information can be obtained from the CHDI Foundation (info@chdifoundation.org)” to the table titles. We updated the title for Table S15 to “Table S15. CAG and CCG repeat sizes for the French cohort. The repeat sizes were quantified by NanoRepeat from Oxford Nanopore long reads.” We updated Table S10 to show that the genomic positions are based on T2T-CHM13v1.1.

In the originally published version of this article, there is a typographical error in the figure panels. In the legend for Figure 5, “(E)” should be “(D),” and “(F)” should be “(E and F).” Additionally, in the main text, the citation of Figure 6I should be Figure 6G.

The results and conclusions of the manuscript are not affected by the above changes. The article has been corrected online. The authors regret the errors.

