# Supplementary material for: Haplotyping SNPs for allele-specific gene editing of the expanded huntingtin allele using long-read sequencing
Source: HGG Adv. 2022 Sep 26;4(1):100146. doi: 10.1016/j.xhgg.2022.100146 (PMC9574884; doi:10.1016/j.xhgg.2022.100146)
Supplement: Document S1. Figures S1–S12 and Tables S1–S3, S5, S7–S9, and S12 [file mmc1.pdf]

Human Genetics and Genomics Advances, Volume ■ ■

Supplemental information

Haplotyping SNPs for allele-specific gene editing of the expanded huntingtin allele using long-read sequencing

Li Fang, Alex Mas Monteys, Alexandra Dürr, Megan Keiser, Congsheng Cheng, Ahkil Harapanahalli, Pedro Gonzalez-Alegre, Beverly L. Davidson, and Kai Wang

Table of Contents

Supplemental Figures 3

Figure S1 3

Figure S2 4

Figure S3 4

Figure S4 6

Figure S5 7

Figure S6 8

Figure S7 9

Figure S8 10

Figure S9 11

Figure S10 12

Figure S11 13

Figure S12 14

Supplemental Tables 15

Table S1 15

Table S2 16

Table S3 17

Table S4 18

Table S5 18

Table S6 18

Table S7 19

Table S8 20

Table S9 21

Table S10 22

Table S11 22

Table S12 22

Table S13 22

Table S14 22

Table S15 23

Table S16 23

Supplemental References....23

Supplemental Figures

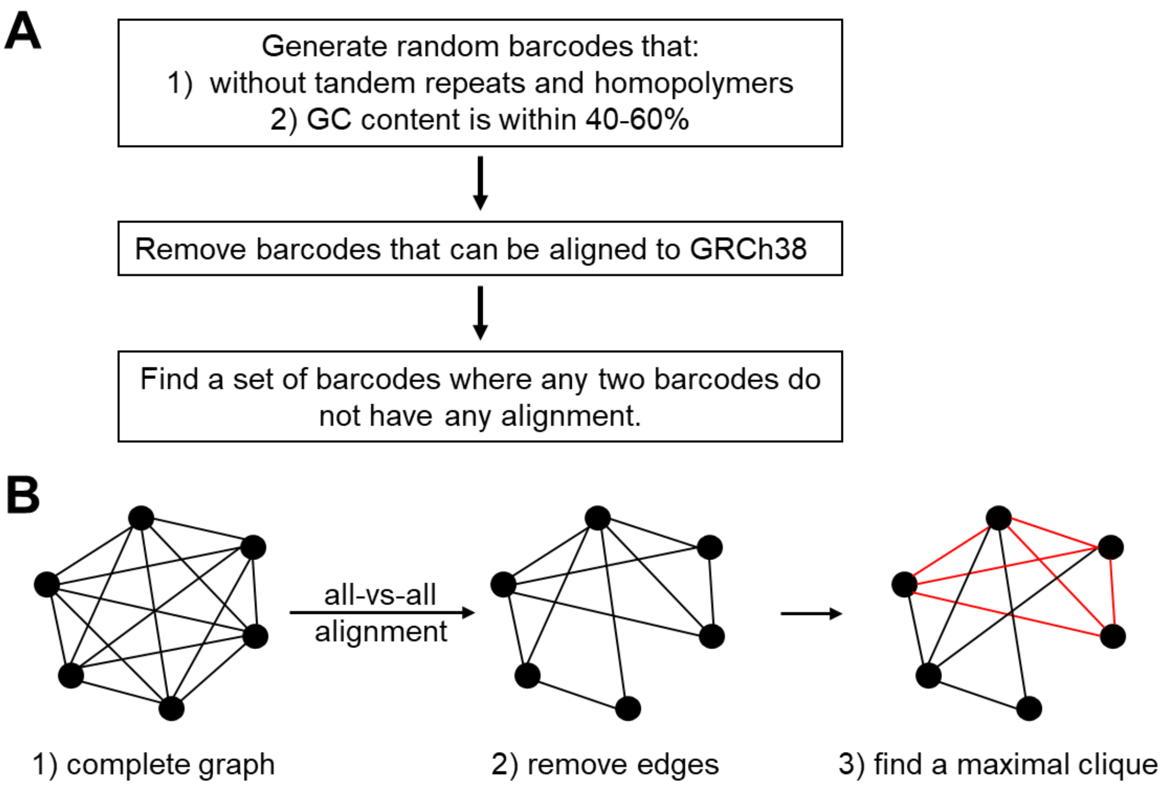

Figure S1

Barcode design strategy used in this study. **A**) The barcode design workflow. **B**) The algorithm to find a set of barcodes where any two barcodes do not have any alignment. 1) Each barcode was a node in the graph. Initially, all nodes are connected in the undirected graph. 2) An all-vs-all alignment of the barcode sequences was performed, and the edge between two barcodes (nodes) was removed if the two barcodes were aligned. 3) The remaining edges only connect barcode pairs that have no alignment. Therefore, a complete subgraph (clique) is a set of barcodes in which any two barcodes have no alignment.

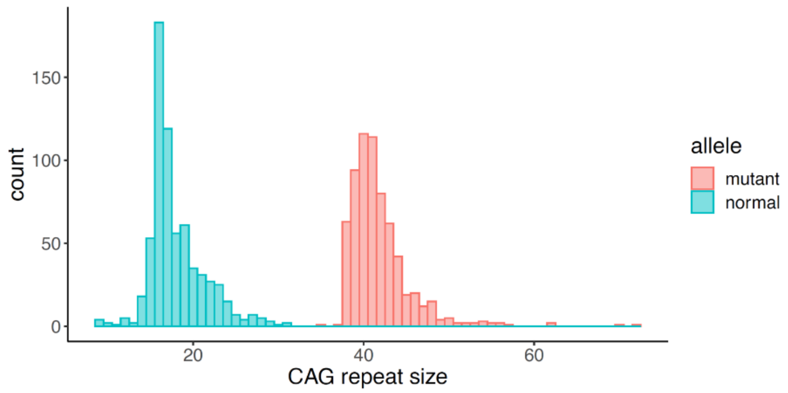

Figure S2

Histogram of the CAG repeat size of the CHDI cohort. The repeat was quantified by AmpRepeat using the Oxford Nanopore long-read sequencing data.

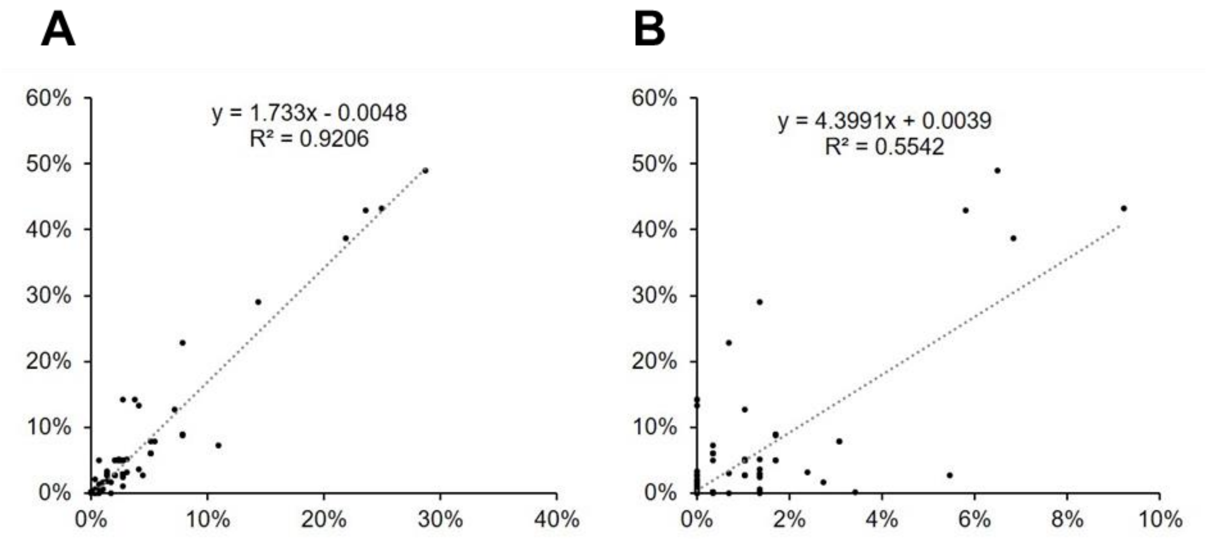

Figure S3

Scatter plots showing the of AFs of SNPs in the CHDI HD cohort (Caucasians) and the gnomAD database (non-Finnish European population). a) normal alleles; b) *mHTT* alleles.

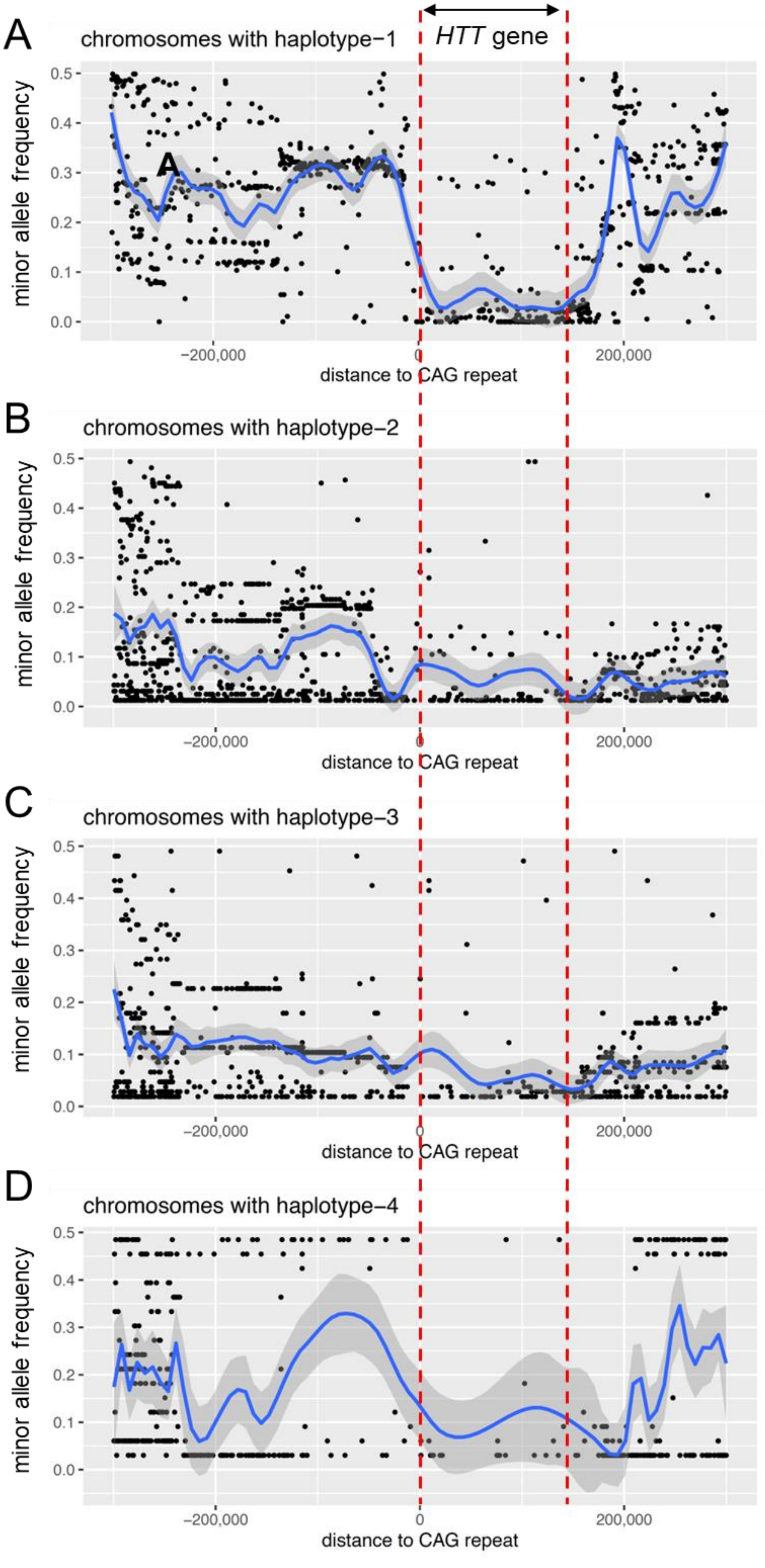

A
chromosomes with haplotype−1
HTT gene
minor allele frequency
distance to CAG repeat
B
chromosomes with haplotype−2
minor allele frequency
distance to CAG repeat
C
chromosomes with haplotype−3
minor allele frequency
distance to CAG repeat
D
chromosomes with haplotype−4
minor allele frequency
distance to CAG repeat

Figure S4

Minor allele frequencies of SNPs in chromosomes with haplotypes 1,2,3, and 4. The data is based on 1000 Genomes individuals (phase 3 data set, non-Finnish European population). The region between dashed red lines is the HTT gene. Minor allele is the allele with frequency ≤ 0.5.

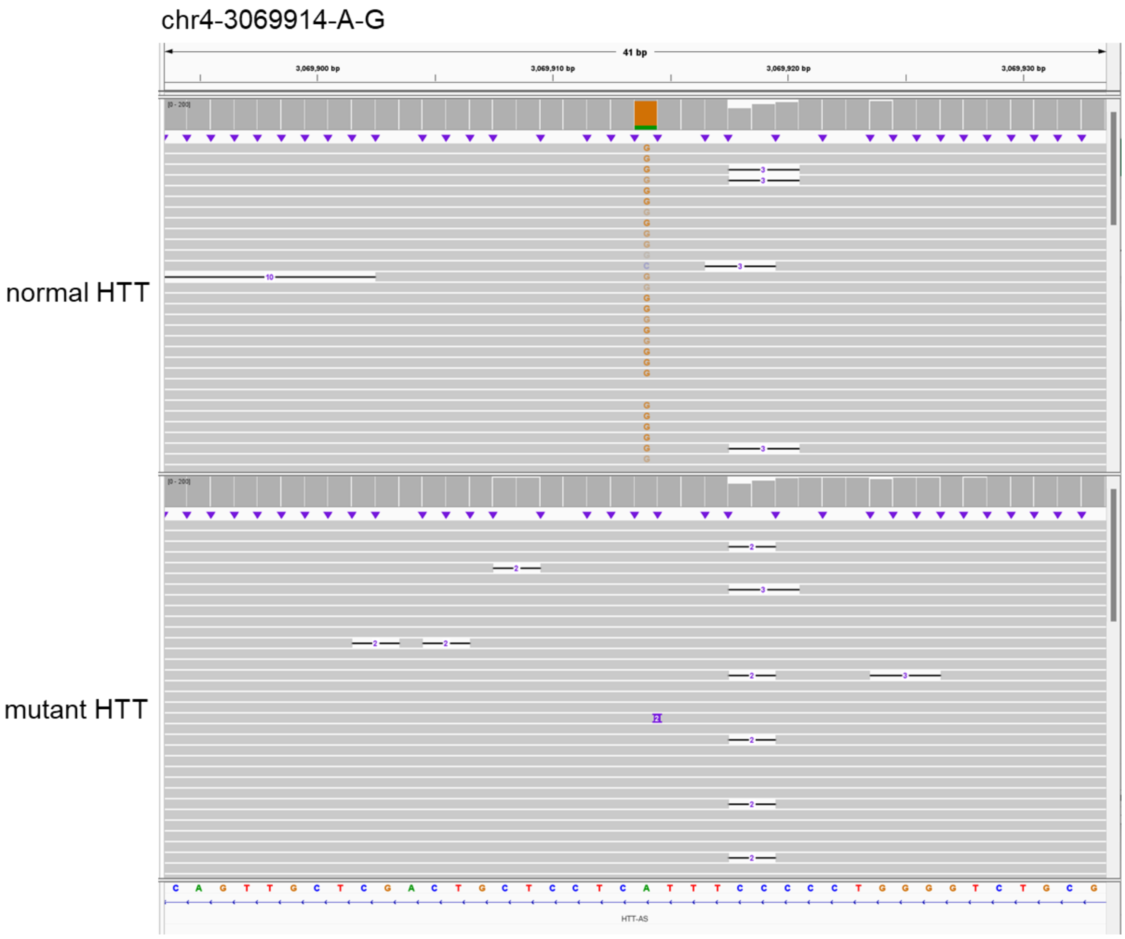

Figure S5

Integrative Genomics Viewer (IGV) showing the aligned sequences around chr4:3069914 (GRCh38). Matched bases are in grey and mismatched bases are colored. The mismatched bases indicate an SNP in the normal HTT but not in the mutant HTT.

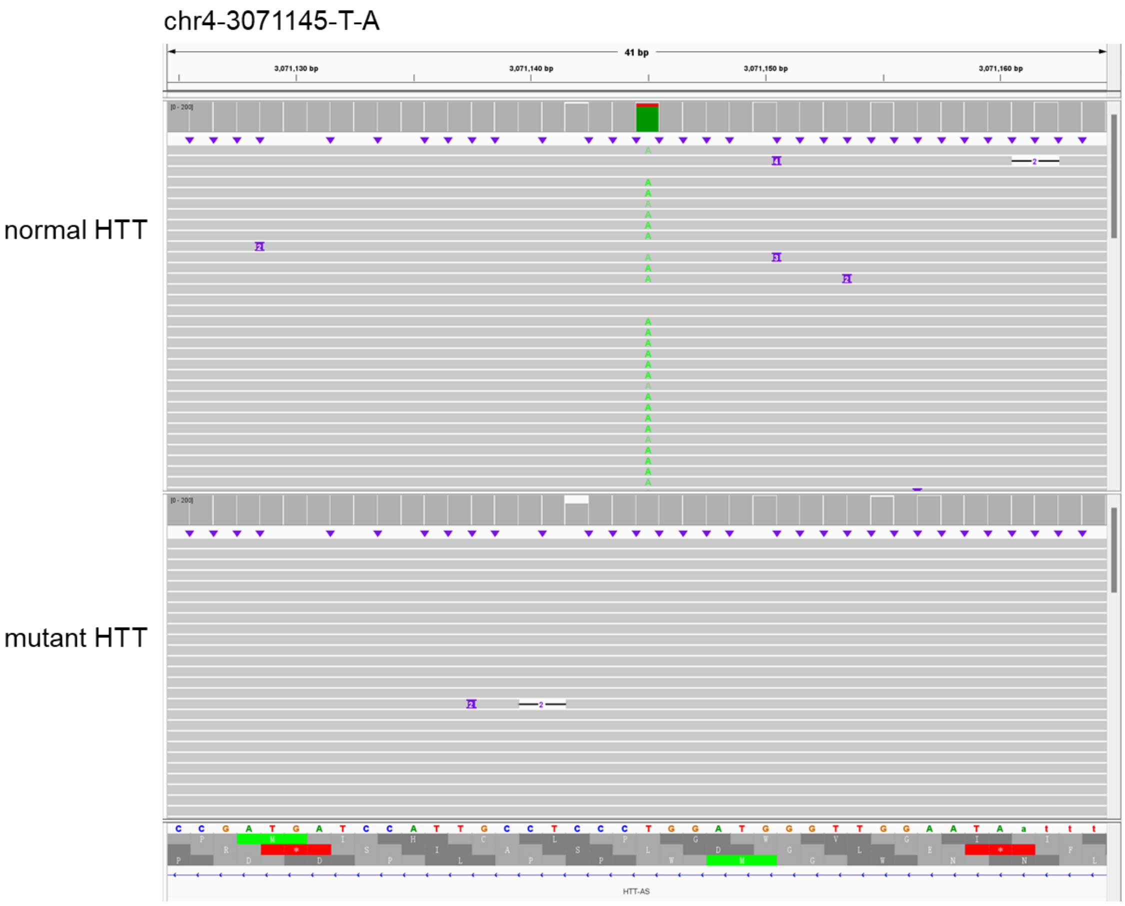

Figure S6

Integrative Genomics Viewer (IGV) showing the aligned sequences around chr4:3071145 (GRCh38). Matched bases are in grey and mismatched bases are colored. The mismatched bases indicate an SNP in the normal HTT but not in the mutant HTT.

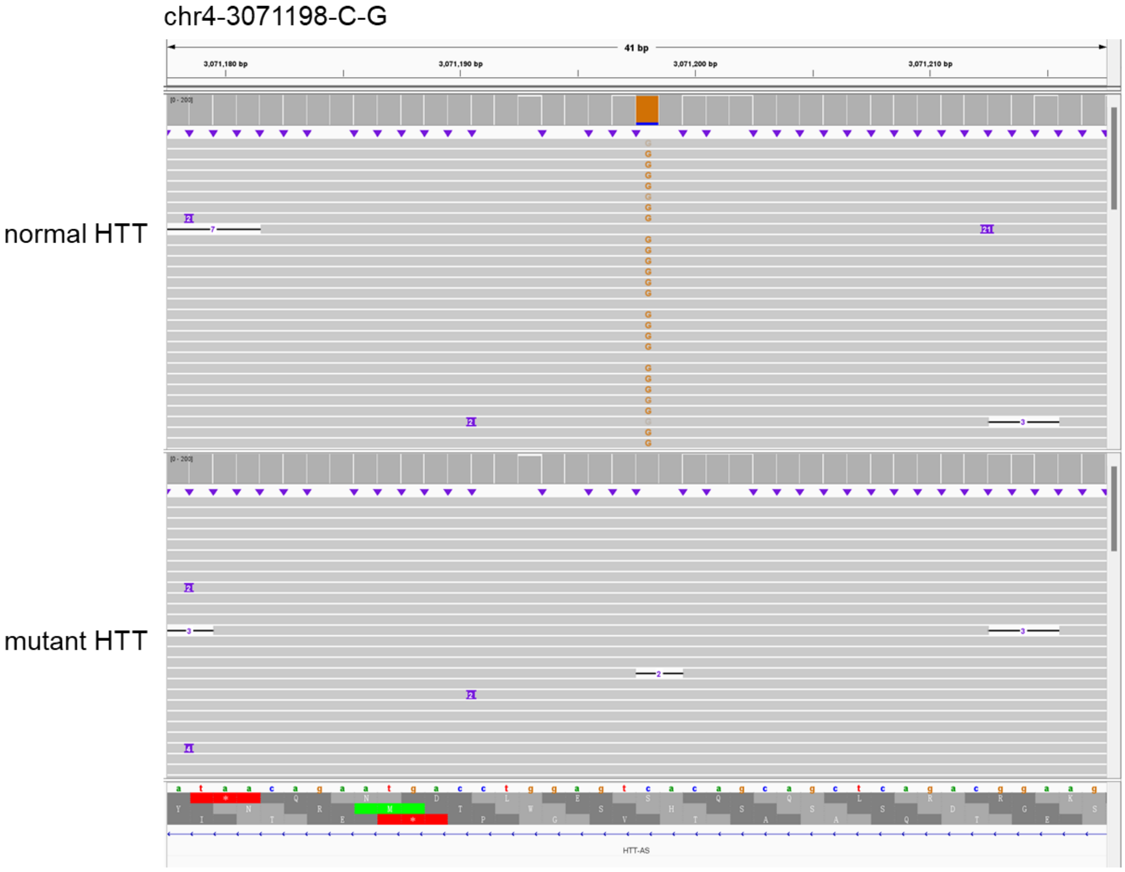

Figure S7

Integrative Genomics Viewer (IGV) showing the aligned sequences around chr4:3071198 (GRCh38). Matched bases are in grey and mismatched bases are colored. The mismatched bases indicate an SNP in the normal HTT but not in the mutant HTT.

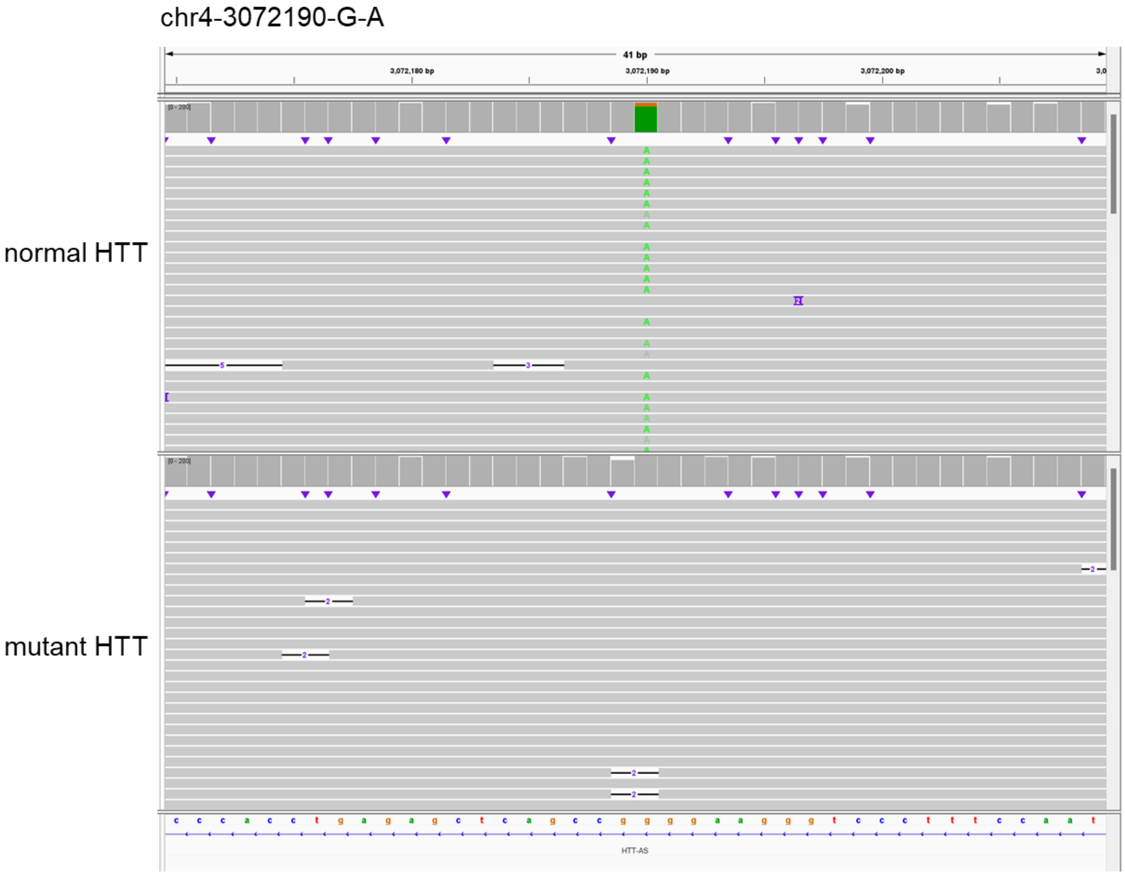

Figure S8

Integrative Genomics Viewer (IGV) showing the aligned sequences around chr4:3072190 (GRCh38). Matched bases are in grey and mismatched bases are colored. The mismatched bases indicate an SNP in the normal HTT but not in the mutant HTT.

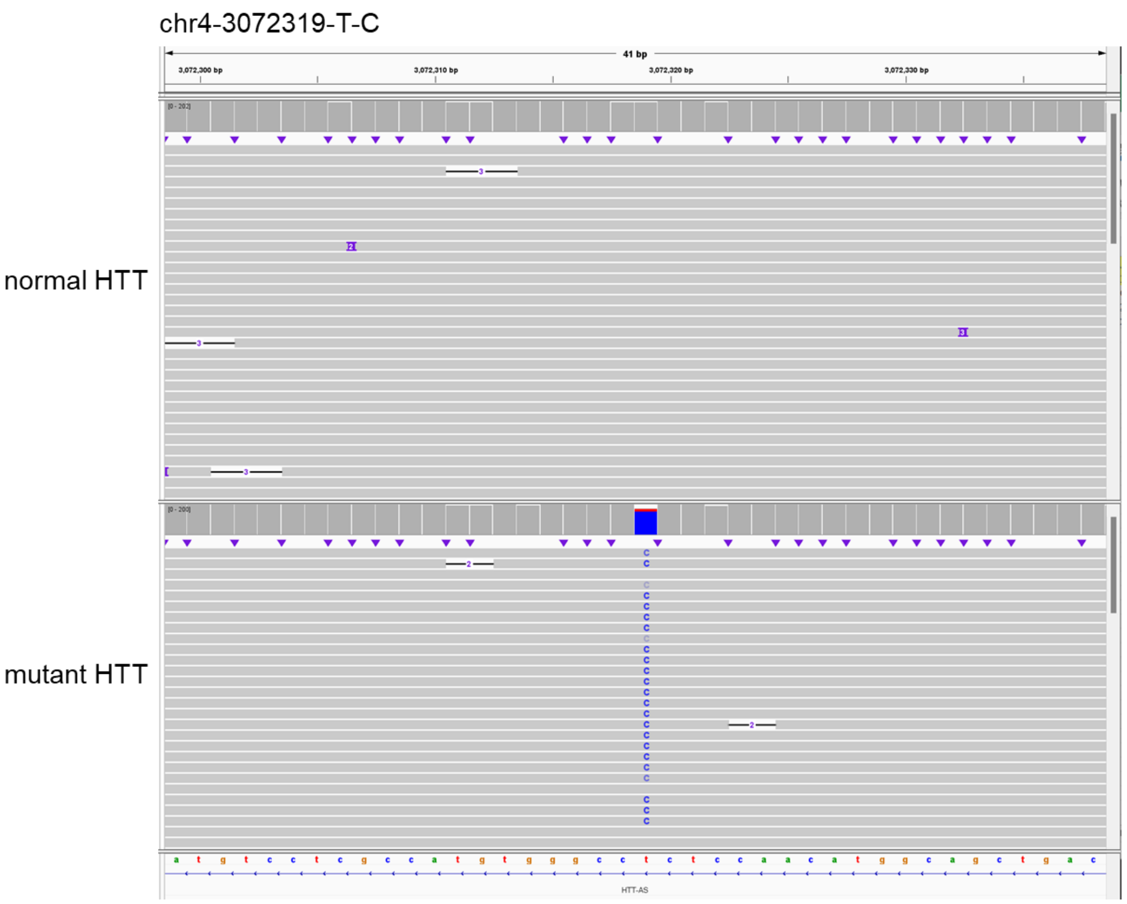

Figure S9

Integrative Genomics Viewer (IGV) showing the aligned sequences around chr4:3072319 (GRCh38). Matched bases are in grey and mismatched bases are colored. The mismatched bases indicate an SNP in the mutant HTT but not in the normal HTT.

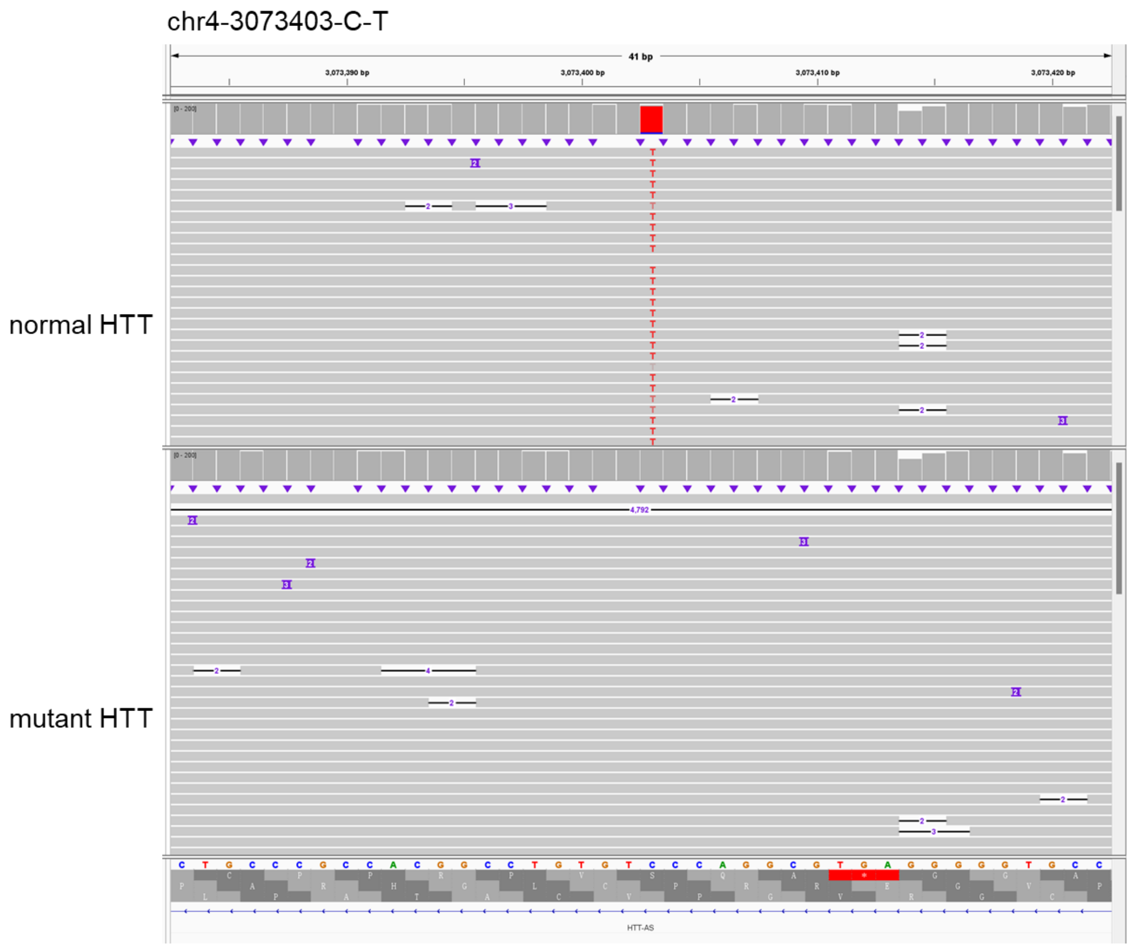

Figure S10

Integrative Genomics Viewer (IGV) showing the aligned sequences around chr4:3073403 (GRCh38). Matched bases are in grey and mismatched bases are colored. The mismatched bases indicate an SNP in the normal HTT but not in the mutant HTT.

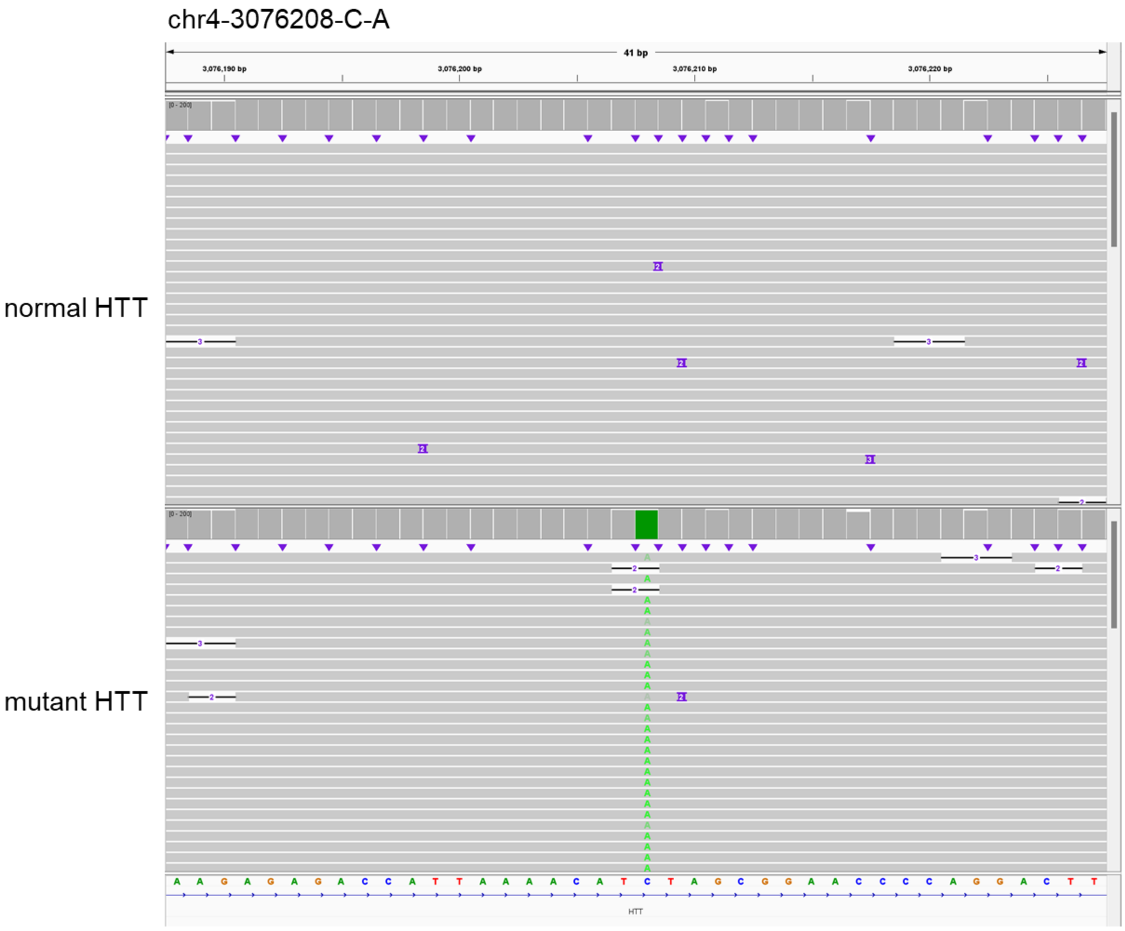

Figure S11

Integrative Genomics Viewer (IGV) showing the aligned sequences around chr4:3076208 (GRCh38). Matched bases are in grey and mismatched bases are colored. The mismatched bases indicate an SNP in the mutant HTT but not in the normal HTT.

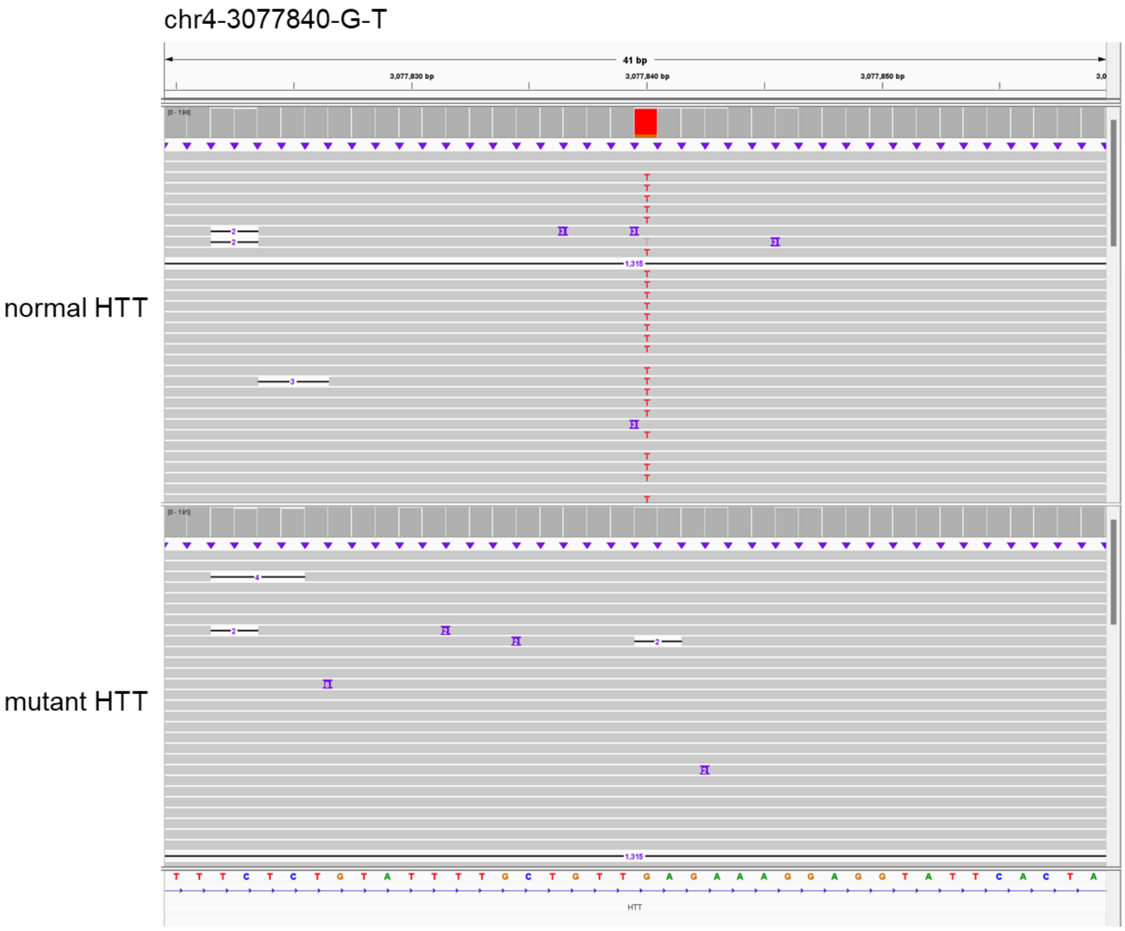

Figure S12

Integrative Genomics Viewer (IGV) showing the aligned sequences around chr4:3077840 (GRCh38). Matched bases are in grey and mismatched bases are colored. The mismatched bases indicate an SNP in the normal HTT but not in the mutant HTT.

Supplemental Tables

Table S1

Barcoded primers for amplicon-1. The left part in the sequence is the barcode.

primer ID	primer sequence	direction
A1B01F	5'-AATTCGCCAGTGATGC-AAAACGAGGGTTGTCAAAGACCCCA-3'	forward
A1B02F	5'-CAGCCATTGATGTCGA-AAAACGAGGGTTGTCAAAGACCCCA-3'	forward
A1B03F	5'-GGCCGCTAGTAATTCA-AAAACGAGGGTTGTCAAAGACCCCA-3'	forward
A1B04F	5'-TCAGGCGCCGATTAAT-AAAACGAGGGTTGTCAAAGACCCCA-3'	forward
A1B05F	5'-ACTAAGCGAGGTCTCT-AAAACGAGGGTTGTCAAAGACCCCA-3'	forward
A1B06F	5'-CGTTCCATCGAGTAAG-AAAACGAGGGTTGTCAAAGACCCCA-3'	forward
A1B07F	5'-ACTGTTCAGACGATCG-AAAACGAGGGTTGTCAAAGACCCCA-3'	forward
A1B08F	5'-CCTCGACGTGGATAAT-AAAACGAGGGTTGTCAAAGACCCCA-3'	forward
A1B09F	5'-GTACATCGGATGATCC-AAAACGAGGGTTGTCAAAGACCCCA-3'	forward
A1B10F	5'-TACGGCGCTATTGAAC-AAAACGAGGGTTGTCAAAGACCCCA-3'	forward
A1B01R	5'-AATTCGCCAGTGATGC-GAGGGAAGTGGCACTGAGCAAATCT-3'	reverse
A1B02R	5'-CAGCCATTGATGTCGA-GAGGGAAGTGGCACTGAGCAAATCT-3'	reverse
A1B03R	5'-GGCCGCTAGTAATTCA-GAGGGAAGTGGCACTGAGCAAATCT-3'	reverse
A1B04R	5'-TCAGGCGCCGATTAAT-GAGGGAAGTGGCACTGAGCAAATCT-3'	reverse
A1B05R	5'-ACTAAGCGAGGTCTCT-GAGGGAAGTGGCACTGAGCAAATCT-3'	reverse
A1B06R	5'-CGTTCCATCGAGTAAG-GAGGGAAGTGGCACTGAGCAAATCT-3'	reverse
A1B07R	5'-ACTGTTCAGACGATCG-GAGGGAAGTGGCACTGAGCAAATCT-3'	reverse
A1B08R	5'-CCTCGACGTGGATAAT-GAGGGAAGTGGCACTGAGCAAATCT-3'	reverse
A1B09R	5'-GTACATCGGATGATCC-GAGGGAAGTGGCACTGAGCAAATCT-3'	reverse
A1B10R	5'-TACGGCGCTATTGAAC-GAGGGAAGTGGCACTGAGCAAATCT-3'	reverse

Table S2

Barcoded primers for amplicon-2. The left part in the sequence is the barcode.

primer ID	primer sequence	direction
A2B01F	5'-CGTCGTTAACAGCGTACAGCCATTGATGTCGA-AAAGTCCCGATGATCCATTGCCTCC-3'	forward
A2B02F	5'-CGATAGTCTTACGAGCGGCCGCTAGTAATTCA-AAAGTCCCGATGATCCATTGCCTCC-3'	forward
A2B03F	5'-GCGAACGATCAGTCTTTCAGGCGCCGATTAAT-AAAGTCCCGATGATCCATTGCCTCC-3'	forward
A2B04F	5'-TGCCATGGCGTATACAACTAAGCGAGGTCTCT-AAAGTCCCGATGATCCATTGCCTCC-3'	forward
A2B05F	5'-AGCGCATCATTGGCATCGTTCCATCGAGTAAG-AAAGTCCCGATGATCCATTGCCTCC-3'	forward
A2B06F	5'-GACGACGTATGTACCTAATTCGCCAGTGATGC-AAAGTCCCGATGATCCATTGCCTCC-3'	forward
A2B07F	5'-ATAAGTTGCGCACGCTACTGTTCAGACGATCG-AAAGTCCCGATGATCCATTGCCTCC-3'	forward
A2B08F	5'-ATAACACGGTCCGGTTCCTCGACGTGGATAAT-AAAGTCCCGATGATCCATTGCCTCC-3'	forward
A2B09F	5'-GGTTAGATTCACGACCGTACATCGGATGATCC-AAAGTCCCGATGATCCATTGCCTCC-3'	forward
A2B10F	5'-AACGGTTCATGAGCCTTACGGCGCTATTGAAC-AAAGTCCCGATGATCCATTGCCTCC-3'	forward
A2B01R	5'-CGTCGTTAACAGCGTACAGCCATTGATGTCGA-ACAAACCTGATAACGCAAGCTACTGC-3'	reverse
A2B02R	5'-CGATAGTCTTACGAGCGGCCGCTAGTAATTCA-ACAAACCTGATAACGCAAGCTACTGC-3'	reverse
A2B03R	5'-GCGAACGATCAGTCTTTCAGGCGCCGATTAAT-ACAAACCTGATAACGCAAGCTACTGC-3'	reverse
A2B04R	5'-TGCCATGGCGTATACAACTAAGCGAGGTCTCT-ACAAACCTGATAACGCAAGCTACTGC-3'	reverse
A2B05R	5'-AGCGCATCATTGGCATCGTTCCATCGAGTAAG-ACAAACCTGATAACGCAAGCTACTGC-3'	reverse
A2B06R	5'-GACGACGTATGTACCTAATTCGCCAGTGATGC-ACAAACCTGATAACGCAAGCTACTGC-3'	reverse
A2B07R	5'-ATAAGTTGCGCACGCTACTGTTCAGACGATCG-ACAAACCTGATAACGCAAGCTACTGC-3'	reverse
A2B08R	5'-ATAACACGGTCCGGTTCCTCGACGTGGATAAT-ACAAACCTGATAACGCAAGCTACTGC-3'	reverse
A2B09R	5'-AACGGTTCATGAGCCTTACGGCGCTATTGAAC-ACAAACCTGATAACGCAAGCTACTGC-3'	reverse
A2B10R	5'-GGTTAGATTCACGACCGTACATCGGATGATCC-ACAAACCTGATAACGCAAGCTACTGC-3'	reverse

Table S3

Barcoded primers for amplicon-3. The left part of the sequence is the barcode.

primer ID	primer sequence	direction
A3B01F	5'-TCGTATCGTGAGCGTCAACCGACTGAGCATAA-AAAACGAGGGTTGTCAAAGACCCCA-3'	forward
A3B02F	5'-TTAGTCACTGTACAGCGTGAGGCGTAGTTCAC-AAAACGAGGGTTGTCAAAGACCCCA-3'	forward
A3B03F	5'-ATCAGTACGTTGCTAGCTTGAGCGATAGCCAG-AAAACGAGGGTTGTCAAAGACCCCA-3'	forward
A3B04F	5'-AGTATGCACGACCGGATCTGTCAACGATACGT-AAAACGAGGGTTGTCAAAGACCCCA-3'	forward
A3B05F	5'-GAAGTCTAGATCAATCGTTAGCATCTGCTCGC-AAAACGAGGGTTGTCAAAGACCCCA-3'	forward
A3B06F	5'-AGACGTCTGACGATGCTCATAACCTGGACATC-AAAACGAGGGTTGTCAAAGACCCCA-3'	forward
A3B07F	5'-CTCATTCGATGTATGTCGCAGGTAGCAAGCAT-AAAACGAGGGTTGTCAAAGACCCCA-3'	forward
A3B08F	5'-TACTGTCGATTCGACCACGACTAGGCTATGCT-AAAACGAGGGTTGTCAAAGACCCCA-3'	forward
A3B09F	5'-CTGTACTCCGATGAACGGCGATCTAGTCTACG-AAAACGAGGGTTGTCAAAGACCCCA-3'	forward
A3B10F	5'-CGATGGTACTCAGATCGGCGACATCAGTTGAT-AAAACGAGGGTTGTCAAAGACCCCA-3'	forward
A3B11F	5'-AGTGCTAGTCGATGCCGCTGCATACCTATGAT-AAAACGAGGGTTGTCAAAGACCCCA-3'	forward
A3B12F	5'-ACATCTTACGGCTCGACTGGCACGATGTCTGA-AAAACGAGGGTTGTCAAAGACCCCA-3'	forward
A3B13F	5'-ACTTAAGTCGAGTCGCATGCCTGTCGCTAGAT-AAAACGAGGGTTGTCAAAGACCCCA-3'	forward
A3B14F	5'-ATCAAGATGTACCACGTCAGGCTAGTACTGCT-AAAACGAGGGTTGTCAAAGACCCCA-3'	forward
A3B15F	5'-TCGAGCTTCGAGTGATAACGTAACGCTGCGTA-AAAACGAGGGTTGTCAAAGACCCCA-3'	forward
A3B16F	5'-GCAGATGACCACTACGTCGAACTGACTTGACT-AAAACGAGGGTTGTCAAAGACCCCA-3'	forward
A3B17F	5'-TCAGCATAGCGTCGATCACCAATGCATGCTAG-AAAACGAGGGTTGTCAAAGACCCCA-3'	forward
A3B18F	5'-CTCGATGACAGATGCGATACTGGCGTTCAATG-AAAACGAGGGTTGTCAAAGACCCCA-3'	forward
A3B19F	5'-GCGTCAGCTACGATTGTATCCAAGTGCTCGAT-AAAACGAGGGTTGTCAAAGACCCCA-3'	forward
A3B20F	5'-GACATTGACTGCTATGACGCCTTGAGTAGCAG-AAAACGAGGGTTGTCAAAGACCCCA-3'	forward
A3B21F	5'-AGCAACGCTAGTGGCCGCTATGTACTAGCTCG-AAAACGAGGGTTGTCAAAGACCCCA-3'	forward
A3B22F	5'-TACATCTGGCGAGTATGATCCTACGGTGAGTC-AAAACGAGGGTTGTCAAAGACCCCA-3'	forward
A3B23F	5'-ATCAGCTGTTACGATAGGCGACTCGCCATCGA-AAAACGAGGGTTGTCAAAGACCCCA-3'	forward
A3B24F	5'-CGCACTAGTATCAGCCTAAGCACTCGTGATGG-AAAACGAGGGTTGTCAAAGACCCCA-3'	forward
A3B01R	5'-TCGTATCGTGAGCGTCAACCGACTGAGCATAA-ACAAACCTGATAACGCAAGCTACTGC-3'	reverse
A3B02R	5'-TTAGTCACTGTACAGCGTGAGGCGTAGTTCAC-ACAAACCTGATAACGCAAGCTACTGC-3'	reverse
A3B03R	5'-ATCAGTACGTTGCTAGCTTGAGCGATAGCCAG-ACAAACCTGATAACGCAAGCTACTGC-3'	reverse
A3B04R	5'-AGTATGCACGACCGGATCTGTCAACGATACGT-ACAAACCTGATAACGCAAGCTACTGC-3'	reverse
A3B05R	5'-GAAGTCTAGATCAATCGTTAGCATCTGCTCGC-ACAAACCTGATAACGCAAGCTACTGC-3'	reverse
A3B06R	5'-AGACGTCTGACGATGCTCATAACCTGGACATC-ACAAACCTGATAACGCAAGCTACTGC-3'	reverse
A3B07R	5'-CTCATTCGATGTATGTCGCAGGTAGCAAGCAT-ACAAACCTGATAACGCAAGCTACTGC-3'	reverse
A3B08R	5'-TACTGTCGATTCGACCACGACTAGGCTATGCT-ACAAACCTGATAACGCAAGCTACTGC-3'	reverse
A3B09R	5'-CTGTACTCCGATGAACGGCGATCTAGTCTACG-ACAAACCTGATAACGCAAGCTACTGC-3'	reverse
A3B10R	5'-CGATGGTACTCAGATCGGCGACATCAGTTGAT-ACAAACCTGATAACGCAAGCTACTGC-3'	reverse
A3B11R	5'-AGTGCTAGTCGATGCCGCTGCATACCTATGAT-ACAAACCTGATAACGCAAGCTACTGC-3'	reverse
A3B12R	5'-ACATCTTACGGCTCGACTGGCACGATGTCTGA-ACAAACCTGATAACGCAAGCTACTGC-3'	reverse
A3B13R	5'-ACTTAAGTCGAGTCGCATGCCTGTCGCTAGAT-ACAAACCTGATAACGCAAGCTACTGC-3'	reverse
A3B14R	5'-ATCAAGATGTACCACGTCAGGCTAGTACTGCT-ACAAACCTGATAACGCAAGCTACTGC-3'	reverse
A3B15R	5'-TCGAGCTTCGAGTGATAACGTAACGCTGCGTA-ACAAACCTGATAACGCAAGCTACTGC-3'	reverse
A3B16R	5'-GCAGATGACCACTACGTCGAACTGACTTGACT-ACAAACCTGATAACGCAAGCTACTGC-3'	reverse
A3B17R	5'-TCAGCATAGCGTCGATCACCAATGCATGCTAG-ACAAACCTGATAACGCAAGCTACTGC-3'	reverse
A3B18R	5'-CTCGATGACAGATGCGATACTGGCGTTCAATG-ACAAACCTGATAACGCAAGCTACTGC-3'	reverse
A3B19R	5'-GCGTCAGCTACGATTGTATCCAAGTGCTCGAT-ACAAACCTGATAACGCAAGCTACTGC-3'	reverse
A3B20R	5'-GACATTGACTGCTATGACGCCTTGAGTAGCAG-ACAAACCTGATAACGCAAGCTACTGC-3'	reverse

A3B21R	5'-AGCAACGCTAGTGGCCGCTATGTACTAGCTCG-ACAAACCTGATAACGCAAGCTACTGC-3'	reverse
A3B22R	5'-TACATCTGGCGAGTATGATCCTACGGTGAGTC-ACAAACCTGATAACGCAAGCTACTGC-3'	reverse
A3B23R	5'-ATCAGCTGTTACGATAGGCGACTCGCCATCGA-ACAAACCTGATAACGCAAGCTACTGC-3'	reverse
A3B24R	5'-CGCACTAGTATCAGCCTAAGCACTCGTGATGG-ACAAACCTGATAACGCAAGCTACTGC-3'	reverse

Table S4

SNP frequencies in normal and mutant HTT. The SNPs are annotated with frequencies in different databases including GnomAD v3.0, GnomAD v2.1.1, and 1000 Genomes Project Phase 3. (in a separate Excel file)

Table S5

CRISPR enzymes and PAMs analyzed in this study.

Enzyme	High efficiency PAMs (included)	Low efficiency PAMs (excluded)	Reference
SpCas9	NGG, NAG	-	-
SpCas9_VQR	NGAG, NGAT, NGAC, NGAA, NGCG, NGTG, NGGG, NAAG	NGGA, NGGT, NGGC	(1)
SpCas9_EQR	NGAG, NGCG, NGAT, NGAA	NGGG, NGTG, NGAC	(1)
SpCas9_VRER	NGCG	-	(1)
SaCas9	NGGGT, NGAAT, NGAGT, NGGAT	NGGAA, NGGAC, NGACT, NGGCA, NGGGA, NGATC, NGGAG, NGATT, NGGTA, NGGTT, NGACA, NGATA, NGGGC, NGGGG, NGAGC, NGACC, NGAAG, NGGCT, NGCAT, NGACG, NGCGT, NGCAC, NGATG, NAAGT, NAGGT, NGGCG, NGTTT, NGGCC, NGCTT, NCAGT, NGCGA, NGGTG, NGGTC, NCGAT, NGCAA, NAGAT, NGTGT	(1)
AsCpf1	TTTN	-	(2)

Table S6

SNPs carried by each haplotype. The genome coordinates are based on GRCh38. '0' indicates the reference allele and '1' indicates the alternative allele. (in a separate Excel file)

Table S7

Upstream SNPs with AF > 20% in the chromosomes with haplotype-1. The analysis was based on the 1000 Genomes Project Phase 3 dataset. AF NFE means the allele frequency of the non-Finnish European population.

Position (GRCh38)	Ref allele	Alt allele	Distance to exon-1	AF NFE	AF NFE with Hap1	AF NFE without Hap1	Ref motif	Alt motif	Enzyme	Strand	Effect on the PAM
3062277	G	A	-12404	36.8%	61.3%	16.3%	GG[C]G	GG[T]G	SpCas9_VRER	negative	loss
3060438	A	G	-14243	48.1%	70.6%	29.5%	TT[T]G	TT[C]G	AsCpf1	negative	loss
3060438	A	G	-14243	48.1%	70.6%	29.5%	C[A]AA	C[G]AA	SpCas9_VQR	positive	gain
3060438	A	G	-14243	48.1%	70.6%	29.5%	C[A]AA	C[G]AA	SpCas9_EQR	positive	gain
3059924	G	T	-14757	51.7%	71.4%	35.4%	TT[G]T	TT[T]T	AsCpf1	positive	gain
3059924	G	T	-14757	51.7%	71.4%	35.4%	T[G]TT	T[T]TT	AsCpf1	positive	gain
3059924	G	T	-14757	51.7%	71.4%	35.4%	[G]TTT	[T]TTT	AsCpf1	positive	gain
3058322	G	C	-16359	50.2%	69.8%	34.0%	GA[C]	GA[G]	SpCas9	negative	gain
3058322	G	C	-16359	50.2%	69.8%	34.0%	A[C]AG	A[G]AG	SpCas9_VQR	negative	gain
3058322	G	C	-16359	50.2%	69.8%	34.0%	A[C]AG	A[G]AG	SpCas9_EQR	negative	gain
3056856	A	G	-17825	47.8%	69.8%	29.5%	CA[A]	CA[G]	SpCas9	positive	gain
3056181	T	C	-18500	52.0%	70.0%	37.0%	C[A]TG	C[G]TG	SpCas9_VQR	negative	gain
3056082	A	G	-18599	54.8%	70.6%	41.7%	T[T]TC	T[C]TC	AsCpf1	negative	loss
3056082	A	G	-18599	54.8%	70.6%	41.7%	GA[A]	GA[G]	SpCas9	positive	gain
3056082	A	G	-18599	54.8%	70.6%	41.7%	A[A]AT	A[G]AT	SpCas9_VQR	positive	gain
3056082	A	G	-18599	54.8%	70.6%	41.7%	A[A]AT	A[G]AT	SpCas9_EQR	positive	gain
3055248	T	G	-19433	54.8%	70.6%	41.7%	TG[T]	TG[G]	SpCas9	positive	gain
3055248	T	G	-19433	54.8%	70.6%	41.7%	G[T]G	G[G]G	SpCas9	positive	gain
3055248	T	G	-19433	54.8%	70.6%	41.7%	TG[T]GAT	TG[G]GAT	SaCas9	positive	gain

Table S8

Downstream SNPs with AF > 20% in the chromosomes with haplotype-1. The analysis was based on the 1000 Genomes Project Phase 3 dataset. AF NFE means the allele frequency of the non-Finnish European population.

Position (GRCh38)	Accession Number	Nearest exon	Distance to exon-1	AF NFE	AF NFE with Hap1	AF NFE without Hap1	Ref motif	Alt motif	Enzyme	Strand	Effect on PAM
3095768	rs28820097	exon-3	20680	39.9%	71.1%	13.8%	TT[C]C	TT[T]C	AsCpf1	negative	gain
3095768	rs28820097	exon-3	20680	39.9%	71.1%	13.8%	AG[G]	AG[A]	SpCas9	positive	loss
3095768	rs28820097	exon-3	20680	39.9%	71.1%	13.8%	G[G]AA	G[A]AA	SpCas9_VQR	positive	loss
3095768	rs28820097	exon-3	20680	39.9%	71.1%	13.8%	AG[G]A	AG[A]A	SpCas9_EQR	positive	gain
3095768	rs28820097	exon-3	20680	39.9%	71.1%	13.8%	G[G]AA	G[A]AA	SpCas9_EQR	positive	loss
3095768	rs28820097	exon-3	20680	39.9%	71.1%	13.8%	AG[G]AAT	AG[A]AAT	SaCas9	positive	loss
3107715	rs10015979	exon-6	32627	40.6%	71.4%	15.0%	TA[A]	TA[G]	SpCas9	positive	gain
3132184	rs363080	exon-17	57096	16.5%	27.2%	7.5%	C[G]AG	C[A]AG	SpCas9_EQR	negative	loss
3142714	rs363107	exon-23	67626	16.8%	28.1%	7.5%	TT[T]A	TT[C]A	AsCpf1	negative	loss
3142714	rs363107	exon-23	67626	16.8%	28.1%	7.5%	T[A]AA	T[G]AA	SpCas9_VQR	positive	gain
3142714	rs363107	exon-23	67626	16.8%	28.1%	7.5%	T[A]AA	T[G]AA	SpCas9_EQR	positive	gain
3150086	rs11731237	exon-26	74998	35.5%	66.8%	9.5%	TT[C]C	TT[T]C	AsCpf1	positive	gain
3150086	rs11731237	exon-26	74998	35.5%	66.8%	9.5%	AG[G]	AG[A]	SpCas9	negative	loss
3150086	rs11731237	exon-26	74998	35.5%	66.8%	9.5%	G[G]AA	G[A]AA	SpCas9_VQR	negative	loss
3150086	rs11731237	exon-26	74998	35.5%	66.8%	9.5%	AG[G]A	AG[A]A	SpCas9_EQR	negative	gain
3150086	rs11731237	exon-26	74998	35.5%	66.8%	9.5%	G[G]AA	G[A]AA	SpCas9_EQR	negative	loss
3158750	rs363146	exon-29	83662	100.0%	100.0%	100.0%	TG[A]	TG[G]	SpCas9	positive	gain
3158750	rs363146	exon-29	83662	100.0%	100.0%	100.0%	G[A]GG	G[G]GG	SpCas9_VQR	positive	gain
3164523	rs9884693	exon-29	89435	38.5%	67.6%	14.3%	TG[G]	TG[A]	SpCas9	positive	loss
3164523	rs9884693	exon-29	89435	38.5%	67.6%	14.3%	G[G]GG	G[A]GG	SpCas9_VQR	positive	loss

Table S9

Estimated miss-classification rate of demultiplexing. Each sequencing run has 95 real samples and 5 blank samples. The mis-classification rate was calculated as the average number of reads in blank samples divided by the average number of reads in real samples.

	Round 1 PCR (16 bp barcode)					Round 2 PCR (32 bp barcode)			
	plate1	plate2	plate3	plate4	plate5	plate 1	plate 2	plate 3	plate 4
number of reads assigned to one of the 100 bins	29872	50653	48452	98692	117591	520487	426535	602341	378664
number of reads assigned to 95 samples	29870	50649	48451	98689	117582	520343	426465	602265	378569
number of reads assigned to 5 blank samples	2	4	1	3	9	144	70	76	95
miss-classification rate	0.13%	0.15%	0.04%	0.06%	0.15%	0.53%	0.31%	0.24%	0.48%
average number of reads per sample	314	533	510	1039	1238	5477	4489	6340	3985

Table S10

The list of filtered STR regions in CHM13 for evaluation of repeat quantification. This list includes all STR regions that are > 100 bp and not within a 500 bp flanking region of another STR. We removed adjacent STRs because many of the adjacent STRs have similar sequences and it is hard to tell if they need to be merged or not without manual examination. Percent_match and percent_indel were calculated by Tandem Repeat Finder (TRF) v4.09. (in a separate Excel file)

Table S11

Detailed information of the CHDI cohort. Race, sex, region, and CAG repeat size (measured by PCR-based Fragment Analysis) of each subject are shown. Subjects are deidentified. This information was provided by the CHDI foundation. (in a separate Excel file)

Table S12

Number of samples of each ethnic group included in the CHDI cohort.

	**# of samples**	**# of QC-passed samples**
American Black	22	16
American Indian	6	5
Asian	6	5
Caucasian	825	610
Hispanic or Latino Origin	73	53
Mixed	18	12
Other	10	7

Table S13

Phased SNPs of each individual in the French cohort (in a separate Excel file). The genome coordinates are based on GRCh38. (in a separate Excel file)

Table S14

Phased SNPs of each individual in the CHDI cohort (in a separate Excel file). The genome coordinates are based on GRCh38. (in a separate Excel file)

Table S15

CAG and CCG repeat sizes for the French cohort. The repeat sizes were quantified by NanoRepeat from Oxford Nanopore long reads. (in a separate Excel file).

Table S16

CAG and CCG repeat sizes for the CHDI cohort. The repeat sizes were quantified by NanoRepeat from Oxford Nanopore long reads. (in a separate Excel file).

Supplemental References

1. Kleinstiver BP, Prew MS, Tsai SQ, Topkar VV, Nguyen NT, Zheng Z, et al. Engineered CRISPR-Cas9 nucleases with altered PAM specificities. Nature. 2015;523(7561):481-5.

2. Zetsche B, Gootenberg JS, Abudayyeh OO, Slaymaker IM, Makarova KS, Essletzbichler P, et al. Cpf1 is a single RNA-guided endonuclease of a class 2 CRISPR-Cas system. Cell. 2015;163(3):759-71.
